# A Review on the High Temperature Strengthening Mechanisms of High Entropy Superalloys (HESA)

**DOI:** 10.3390/ma14195835

**Published:** 2021-10-06

**Authors:** Malefane Joele, Wallace Rwisayi Matizamhuka

**Affiliations:** Department of Chemical and Metallurgical Engineering, Vaal University of Technology, Vanderbijlpark 1911, South Africa; wallace@vut.ac.za

**Keywords:** strengthening mechanisms, high entropy superalloys, solid solution, configuration entropy, thermodynamic of phase stabilities

## Abstract

The studies following HEA inceptions were apparently motivated to search for single-phase solid solution over intermetallic phases, accordingly made possible by the concept of high configurational entropy. However, it was realised that the formation of intermetallic phases in HEAs is prevalent due to other criterions that determine stable phases. Nonetheless, recent efforts have been directed towards attributes of microstructural combinations. In this viewpoint, the techniques used to predict microstructural features and methods of microstructural characterisation are elucidated in HESA fields. The study further analyses shortcomings regarding the design approaches of HESAs. A brief history is given into how HESAs were developed since their birth, to emphasize the evaluation techniques used to elucidate high temperature properties of HESAs, and the incentive thereof that enabled further pursuit of HESAs in the direction of optimal microstructure and composition. The theoretical models of strengthening mechanisms in HEAs are explained. The impact of processing route on the HESAs performance is analysed from previous studies. Thereafter, the future of HESAs in the market is conveyed from scientific opinion. Previous designs of HEAs/HESAs were more based on evaluation experiments, which lead to an extended period of research and considerable use of resources; currently, more effort is directed towards computational and theoretical methods to accelerate the exploration of huge HEA composition space.

## 1. Introduction

In 2004, Yeh et al. [1] and Cantor [2] independently introduced a novel alloy design which caught the limelight in the field of engineering and science. This alloy design differed significantly with the traditional alloy design employed since the time of the Bronze Age and the period thereafter. It has been coined with the name High Entropy Alloy (HEA) by virtue of its high configurational entropy caused by presence of more than 5 elements in almost equal concentration. The reason that the potential of HEA alloys has been realised relatively late, and even overlooked during 20th century, is that in terms of the established Hume-Rothery solid solution rules and phase graphs, multi-component nature of HEAs may result in many phases and ordered compounds, which may in turn produce complex and detrimental microstructures that are difficult to engineer and study. However, Yeh and co-workers [1], using thermodynamic principles, defied this conventional thinking by showing that the presence of five or more components in near equal composition would increase the configurational entropy of mixing sufficiently to deter the enthalpies of compound formation. Thus, simple solid solution phases such as FCC, BCC or HCP will be prominent in these multi-component alloys, and impressive properties might be realised. Cheng et al. [3] argued, on the other hand, that a high mixing entropy effect is not the sole criteria contributing to the stability of solid solution phases in HEA alloys, as variables such as mixing enthalpy, atomic size difference, and valence electron concentration (VEC), must also be considered. Authors further elucidated that the effect of high mixing entropy on the stability of simple solid solution phases decreases with a decrease in temperature, according to Gibbs free energy equation, thus implying the formation of intermetallic phases at relatively low temperatures. Nonetheless, the presence of intermetallic phases can enhance the properties of HEA alloys if efforts are directed towards attributes of microstructural combinations.

HEAs are a new area of interest in research because of their unexplored scope and promising unique combination of properties. However, formidable hurdles of high throughput alloy evaluations need to be overcome to fully exploit their potential in areas of science and engineering. HEAs have given birth to one of its derivatives called High Entropy Superalloys (HESAs), named after their microstructural resemblance to superalloys used in high temperature applications. Few studies have explored the mechanistic design approach to tune high temperature mechanical properties of HESA. In most cases, the studies involve high temperature microstructural stabilities [4,5,6,7,8,9,10,11,12], room temperature mechanical properties [13,14,15,16,17,18,19,20,21,22], and the structure and fraction of the matrix phase and precipitate phase in comparison to well-known Ni-based superalloys [11,23,24,25,26,27,28]. Praveen and Kim [29] revealed that HESAs are regarded as potential materials to be used under a high temperature based on constant strain rate experiments at low temperatures and under the assumption of diffusion being sluggish. The authors argue that the measure of HESA potential as high temperature material should also include creep behaviour at high temperatures. They point out that of the few studies that study the creep behaviours of HESAs, the majority use nanoindentation techniques at room temperature. The drawback with the nanoindentation technique is that it utilises a relatively low strain; as a result, caution must be exercised in the analysis of the data. Detailed evaluation of high temperature mechanical properties to fully exploit HEAs with its multifariousness is still outstanding, Miracle and Senkov [30] mentioned that there are about 171 million possible alloy systems with three to six principal constituents, and only about 122 alloys have been made since the inception of higher-order systems. This means that the chance of missing the best alloy is relatively 99.99993% [30]. To fully exhaust the possible composition and properties of HEA/HESA, the underlying mechanisms that control performance at the macro scale must be fully understood and rapid alloy evaluations must be designed.

Great attempts have been made to explore the multi-dimensional composition space of HEAs/HESAs using computational techniques such as empirical correlations and thermodynamics of phase stabilities. George et al. [31] mentioned this, due to huge compositional space offered by HEAs being quite a hurdle to efficiently probe their microstructural features. Shivam et al. [10] noted that temperature-defined transitions in HEAs do not exist as in bulk-metallic glass; as a result, it imposes a difficulty in designing optimal microstructures in these alloys. Classical thermodynamics have been efficiently used to determine the fraction of possible binary phase diagrams, and a meagre fraction of ternary phase diagrams has also been determined; however, the same fraction decays exponentially as the number of components increases. Thus, there is no definite determined quinary phase diagram due to requirements for higher dimensional representation. Shivam et al. [10] elucidated this using the following analogy: in binary systems, the eutectoid is an invariant point and the free-energy-composition diagram for spinodal decomposition is an inverted bell-shaped curve, whereas in ternary systems, binary eutectic is a line, ternary eutectic is an invariant point and the solid solution is a surface, whilst in quinary systems, quinary eutectic is an invariant point, quaternary eutectic is a line, ternary eutectic is a surface and binary eutectic is a volume. The free-energy composition diagram for ternary spinodal is an inverted three-dimensional bell, while the shape of the quaternary and quinary cannot be determined due to higher dimensional requirements. Therefore, stable phases and their compositions cannot be predicted precisely. As mentioned by Shivam et al. [10], either trial and error experimentation or development of suitable models will perhaps solve this impasse in HEAs. The drawback with trial-and-error experimentation noted by Zhang et al. [32] is that it leads to a high consumption of human and material resources, research cycle extension and low efficiency. The empirical correlation uses the established Hume-Rothery rules and thermodynamic parameters as guides to predict the type of phases in alloys. They are based on composition-weighted terms for differences in parameters such as atomic size misfits, valence electron concentration (VEC), electronegativity and mixing enthalpy [30]. The empirical method’s critical limitation is that they suffer from oversimplification [33]. 

**Figure 1:**
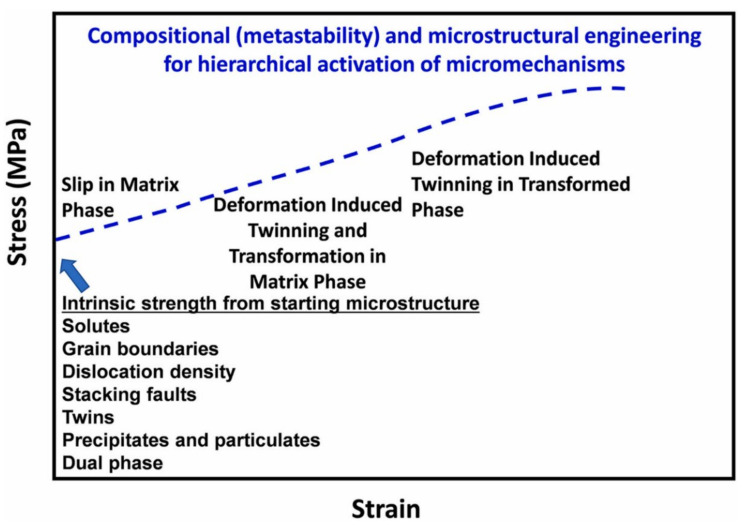
Hypothetical stress-strain curve of HEAs’ design approach by employing micromechanisms induced by composition and structure. Adapted from [37].

Semi-empirical CALPHAD (calculation of phase diagrams) is an approach involving thermodynamics of phase stabilities. It provides an effective and efficient way to map multicomponent phase diagrams by doing mathematical interpolation and extrapolation of the Gibbs energy function of each phase in the composition, temperature and pressure space [33]. However, a set of accurate thermodynamic databases of phases is a prerequisite for predicting and revealing the precise relationships between processing, structure, property and performance [34,35]. George et al. [31] discussed the techniques of truly-ab-initio calculations and CALPHAD, and the authors mentioned that truly-ab-initio calculations are tedious at finite temperatures and are only suitable to screen narrow composition ranges that are of interest and when prediction of phase stabilities need to be accurate, while CALPHADs are based on the development of thermodynamic functions that are an empirical fit to experimental data from binary and ternary phase diagrams. Alas, quaternary and higher-order compositions cannot be used because of the requirement for higher order interactions, which become negligible and weak, and thus a reliable account of HEAs is inferred by a combination and extrapolation of binary and ternary data [30]. Thermodynamic databases, such as TCHEAS, have been developed, which include all binary and as many and near complete ternary systems [33]. The CALPHAD method, as noted by [30], can reliably predict the number and type of phases; however, its predictions are less accurate in terms of transformation temperatures, volume fractions and compositions of phases. As conveyed by Goerge et al. [31], the CALPHAD method is based on thermodynamics, and as a result it may fail to recognise metastable, microstructural constituents and transient phases that are significant in engineering alloys. Manzoni and Glatzel [36] explained that an equilibrium state must be approached in order to establish a database of phases; alas, there is no standard heat treatment that ensures that the respective HEAs form equilibrium microstructures. Other techniques that can be used to determine phase diagrams in HEAs and screen physical and mechanical properties involves combinatorial experimental approaches, as coined in the review by George et al. [31] The author explained that the method involves high-throughput synthesis and metallographic or XRD characterization of miniature samples, which are then used to yield important details about the phases and microstructures present. The drawback with high throughput combinatorial experiments is that it is ineffective in determining many properties of the structural materials, because they depend sensitively on sample dimensions and microstructural length, while the technique miniaturises the sample. Another technique is a mechanism-based, alloy design approach. It is based on the concept of the material’s response to the load using specific strengthening mechanisms, which depends on temperature, stress and strain rate [31]. The authors noted that traditional alloys, such as stainless steels and TWIP, are mechanistically designed using mechanisms of solid solution and precipitation strengthening, deformation twinning and the formation of ε-martensite and α-martensite phases. This design approach also consists of identifying and tuning the thermodynamic and structural parameters that govern the alloys. The mechanistic design approach rules are thus applicable to HEAs with added advantages because of the alloys’ characteristics, such as massive, solid solution strengthening. Therefore, the approach might give birth to several types of new HEA classes. Figure 1 below shows hypothetical stress-strain curve wherein the micromechanisms are induced by composition and structural engineering, which essentially given reliable measure can generate high strength alloys. This review places a focus on mechanistic design approach for HESAs.

Characterisation techniques employed in the field of HEAs to study microscopic details seem to be successful when lots of methods and experimental equipment are integrated. As explained in the review paper by Manzoni and Glatzel [36], solid solution microstructures of HEAs can be inferred by simply indexing X-ray diffraction peaks; however, the method reveals only disordered phases and cannot reveal local distortions. Thus, additional methods, such as synchrotron X-ray and/or neutron diffraction, are needed to further clarify details of atomic structures. Due to the nature of HEA alloys, which tend to have complex structures with multiple phases, especially in as-cast conditions, phase-specific elemental analysis must be part of the microscopic study for these alloys. This is accomplished by methods including EDX (energy dispersive X-ray) and EBSD (electron backscatter diffraction), which are SEM (scanning electron microscope)-based techniques, and SAED (selected area electron diffraction), which is a TEM (transmission electron microscope)-based technique. Manzoni and Glatzel [36] noted that the atomic-level features are beyond the resolution of many techniques. Nonetheless, the specialised techniques such as TEM-based HAADF (high-angle annular dark field) imaging, APT (atom probe tomography), 4D scanning electron microscopy and field ion microscopy, can capture atomic-level details. George et al. [31] wrote that APT gives good near-atomic-scale resolution at a fair precision, but it tends to suffer from aberration deficiencies, lack of structural resolution and overlaps in the peaks detected because different ions can have the same mass-to-charge ratio. While field ion microscopy can accurately provide a single atomic position, it lacks quantitative chemical sensitivity. Gao et al. [38] mentioned that advanced understanding of HEA should include features at several length scales and microstructural characterisation in three-dimensions, as well as the study of behaviours under a wide range of temperatures and environmental conditions. Thus, high resolution analyses of HEAs will require an improvement of the characterisation tools in terms of the features such as detectors and method layout and simulation-enhanced and machine-learning-enhanced image analysis techniques [31]. The technology involving in situ structural and mechanical characterisations at wide temperature ranges exist and must be advanced to uncharted conditions. The drawback in this regard, as noted by Gao et al. [38], is that advanced experimental characterisations tend to be quite difficult and require considerable resources. Nonetheless, the most significant undertaking should rely on relating information gained from high-resolution imaging at the atomic scale into a description of material behaviour under loads and external impetus.

The study therefore considers all the drawbacks regarding the research of HESA in terms of uncharacterised features, which consequently causes a lack of clear modelling of the strengthening mechanisms and purposeful control of microstructures and compositions to enhance mechanical properties, especially under the uncharted high temperature conditions.

## 2. The Development of High Entropy Superalloys

The conventional superalloys have reached a limit in terms of their homologous temperature; as a result, efforts have been directed to improving its cost performance by reducing the use of expensive elements such as rhenium (Re) [39]. The composition space design of conventional superalloys is limited and can only be manipulated to reduce cost and density. Consequently, this calls upon another alloy design for better performance at high temperatures. The solution currently relies on HEAs, which allow the exploitation of a vast composition space. Among HEAs, HESAs are regarded as potential alloys that possess high temperature properties to exceed the temperature limit of conventional superalloys. The reason behind their potential is that they have a microstructural resemblance to conventional superalloys wherein precipitation hardening is guaranteed; and in addition, they possess intrinsic characteristics such as sluggish diffusion and lattice distortion, which makes them thermally stable to maintain such microstructures at high temperatures. There are two types of HESAs viz. refractory high entropy superalloys and 3d-transition-metal (3d-TM) high entropy superalloys. Miracle et al. [39] note the basic microstructural feature of refractory HESAs and 3d-TM HESAs as having a high-volume fraction of discrete and ordered particles incorporated in a thin and continuous channel of a disordered matrix phase. The disordered matrix and ordered particles in refractory HESAs are made up of a BCC (A2) structure and a B2 structure respectively, while in the 3d-TM, HESAs are made up of an FCC (A1) structure and an L12 structure, respectively. Another defining feature of refractory HESAs and 3d-TM HESAs is based on composition [39]. Refractory HESAs contain two or more refractory metals; however, there is no definite classification of refractory metals, using melting temperature as a measure, and metals with a melting temperature above 2200 °C are regarded as refractory metals, although metals with melting temperature as low as 1850 °C are also considered as refractory metals, while 3d-TM HESAs contain dominantly 3d transition metals. Below are the studies that show the development of HESAs from their inception.

As mentioned by Miracle et al. [39], the first superalloy-like microstructure of refractory high entropy alloys was discovered four years after the first design of refractory HEAs and refractory CCAs in 2010. A paper by [40] appears to be the first to produce refractory HEAs with A2 + B2 microstructures resembling that of conventional superalloys. The alloy had a composition of AlMo0.5NbTa0.5TiZr and it was derived from 1st generation refractory HEAs (CrMo0.5NbTa0.5TiZr) that had bcc phase + laves phase. Thus, an ordered second phase particle (B2) was induced by replacement of Cr with Al. AlMo0.5NbTa0.5TiZr alloy was produced by vacuum arc melting and thereafter was remelted five times to obtain homogeneity. After melting, it was subjected to hot isostatic pressing at 1673 K under 207 MPa for 2 h and then annealed at 1673 K for 24 h. In comparison to its parent alloy, AlMo0.5NbTa0.5TiZr showed improved hardness (5.8 MPa) and low density (7.4 g/cm^3^). The two phases in AlMo0.5NbTa0.5TiZr alloys are likely coherent and are at a near equal volume fraction. The authors reported yield strength, maximum strength, elastic modulus, and fracture strains at room temperature to be 2000 MPa, 2368 MPa, 178.6 GPa and 10%, respectively. At high temperatures of 1273 K and 1473 K, yield strength decreased to 745 MPa and 250 MPa, respectively, while compression ductility increased above 50%. In the following papers, Senkov et al. [40,41] explored the effect of Al in stimulating or suppressing formation of intermetallic phase in refractory HEAs. The authors studied six refractory HEAs and showed that only three alloys contained two BCC phases, according to XRD analysis, with no superlattice peaks to suggest ordered phases. The alloys containing two BCC phases are AlMo0.5NbTa0.5TiZr, Al0.3NbTaTi1.4Zr1.3 and Al0.5NbTa0.8Ti1.5V0.2Zr and are denoted A(1), A(4) and A(6), respectively. The two phases of these alloys exist in a form of interpenetrating nanolamellae, creating a basket-weave nanostructure inside grains. A(1) was studied previously as aforementioned. A(4) was derived from Al0.4Hf0.6NbTaTiZr by replacing heavy elements such as Hf with lighter ones viz. Ti and Zr. Al0.4Hf0.6NbTaTiZr alloy is a modified version of 1st generation refractory HEA (HfNbTaTiZr containing single bcc phase) and is part of this study and the previous. A(6) was derived from Al0.3NbTa0.8Ti1.4V0.2Zr0.3 alloy, which is the modified version of A(4). A(6) was founded by partial substitution of Zr with Al and its intermediate Al0.3NbTa0.8Ti1.4V0.2Zr0.3 (part of this study) cousin was founded by an attempt to reduce density, where Ta was replaced with lighter V. A(1) underwent the same processing route as in the previous study, while A(4) and A(6) were subjected to just a change in temperature, viz. 1200 °C, in both hot isostatical pressing and annealing. The reported density of A(4) and A(6) are 8.2 g/cm^3^ and 7.4 g/cm^3^ respectively, while Vicker’s hardness is 4.8 GPa and 5.2 GPa respectively. The yield strength and maximum strength of A(4) and A(6) are improved as compared to their 1st generation refractory HEA parents. Compression ductility of A(4) and A(6) is only 5% at room temperature. All six alloys studied by Senkov et al. [40] have a room temperature specific yield strength considerably higher than Ni-based superalloys. At 800 °C, all studied alloys, except A(4), have specific yield strengths that are higher than IN718 superalloy, and on the other hand A(1) and A(6) are stronger than Mar-M247 superalloys. Although these refractory HESAs have good strength at both room and elevated temperatures, they suffer very limited room temperature compressive ductility, attributed to inherent brittleness of the ordered B2 matrix. Another factor which decreases ductility at room temperature of refractory HESAs is the inverted microstructure, i.e., the ordered B2 phase is the matrix and the disordered phase A2 is the precipitate [42]. Soni et al. [43] enhanced the ductility of Al0.5NbTa0.8Ti1.5V0.2Zr alloy by solving the issue of intrinsic inverted microstructures found in refractory HESAs by reversing the common BCC + B2 microstructure, while maintaining excellent room and a high temperature yield strength. To achieve such a microstructure, the alloy was subjected to heat treatments composed of different conditions. The first condition consists of hot isostatic pressing of the cast alloy and then homogenization at 1200 °C for 24 h, followed by cooling at 10 °C/min. Th second condition consists of creating a solution at 1400 °C for 20 min followed by water quenching. The third condition involves annealing at 600 °C for 120 h followed by water quenching. The resulting microstructure consists of a BCC + B2 phase wherein BCC forms a continuous matrix phase, with an average grain size of ~150 μm. It was revealed that the B2 phase is enriched with Al and Zr, while the BCC phase is enriched with other elements. The alloy showed a good combination of high yield strength and ductility viz. room temperature and the 600 °C yield strength is 1345 MPa and 1423 MPa, respectively, and compressive ductility at room temperature exceeded 20%. The significant note to take home is that the alloy that underwent third processing conditions exhibit a high strength at 600 °C above room temperature. The author recommended a further exploration for this phenomenon. Senkov et al. [44] studied the modified composition of a previously studied AlMo0.5NbTa0.5TiZr alloy in a 1400 °C annealed condition. They found that the alloys with reduced Al viz. Al0.5Mo0.5NbTa0.5TiZr and Al0.25NbTaTiZr retain a two-phase nanostructure; however, the nano-scale precipitates coarsened during deformation and the BCC phase became continuous, while the formerly continuous B2 channels broke into separate elongated particles. The ductility of both alloys considerably increased at 1000 °C. Soni et al. [45] developed a new low-density Al10Nb15TaTi30Zr40 refractory HESA, exhibiting a nano-scale BCC + B2 mixture. This alloy was based on the composition of the B2 phase in a two-phase BCC + B2 mixture of a previously studied Al0.25NbTaTiZr after annealing at 1000 °C for 2 h. The reason for pursuing such a composition is that this refined two-phase microstructure resulted in an excellent combination of room temperature compressive ductility and strength. The alloy was subjected to homogenization at 1100 °C for 24 h followed by water quenching or slow cooling (20 °C/min). Compressive yield strength and ductility at room temperature reached 1075 MPa and 0.55 (true strain at failure), respectively. The alloy shows the highest room temperature compressive ductility compared to the previous study of refractory HESAs. This is attributed to a nanometer-level co-continuous mixture of BCC + B2 phases and a low anti-phase boundary energy in the B2 phase. Therefore, the studies showed that refractory HESA mechanical properties have been gradually improved by manipulations of the processing conditions and composition changes. Thus, a clear relation of underlying structure and properties will further enhance these types of alloys.

Yeh et al. [46] studied the Co1.5CrFeNi1.5Ti0.5 alloy and showed the presence of γ’ particles below 800 °C. This is the first paper to report 3d-TM HEAs with a microstructure similar to that of conventional superalloys. The Co1.5CrFeNi1.5Ti0.5 alloy was first designed by Chuang et al. [47] from the AlxCo1.5CrFeNi1.5Tiy HEA family, and the aim of the paper was to investigate the microstructure and wear behaviour of the alloy with varying contents of Al and Ti. The Co1.5CrFeNi1.5Ti0.5 alloy was designed with the intention of retaining the FCC structure as the primary phase, which meant increasing the strong FCC formers, such as Co and Ni. Chuang et al. [47] found that the Co1.5CrFeNi1.5Ti0.5 alloy consists of a (Co,Cr,Fe)-rich matrix and white blocky precipitates (510μm in size) in interdendritic regions. EDS analysis revealed that precipitates have a composition similar to the η-(Ni,Co)3Ti phase. However, it is noticeable that minor peaks in the XRD pattern could not be characterised, and thus it was assumed to represent some unknown minor phases. Yeh et al. [46] identified the presence of nanosize γ’ particles in an FCC matrix, which are assumed to be unidentified minor peaks in the previous study. The γ’ particles are reported to have compositions similar to those of the η phase, and their presence was confirmed by TEM and SEM analysis. Daoud et al. [48] studied an Al8Co17Cr17Cu8Fe17Ni33 alloy designed to have FCC as its solid solution phase, through increasing the Ni content at the expense of Al and Cu. The analysis of the microstructure on as-cast and heat-treated samples revealed the presence of γ’ precipitates in the FCC matrix and grain boundaries. The precipitates were found to be enriched in Ni, Al and Cu. Heat treatment at 1150 °C for 5 h resulted in a small precipitate size (<10 nm). The tensile strength values at room temperature and 500 °C of the alloy specimens are low compared to IN617 and CMSX-4 superalloys. This was attributed to the small size of the L12 precipitates in this alloy as compared to Ni-based superalloys. Manzoni et al. [49] improved γ’ precipitates of Al8Co17Cr17Cu8Fe17Ni33 alloys by adding small amounts of γ and γ’ stabilizers, such as Mo, Ti and W, using the concept of Ni-based superalloys. The new alloy composition viz. Al8Co17Cr14Cu8Fe17Ni34.3W0.1Mo0.1Ti1 after homogenization at 1250 °C for 80 min and then annealing at 700 °C for 24 h showed precipitates with a size above 50 nm. Pickering et al. [50] showed the formation of the FCC phase and L12 precipitates in the as-cast state and the aged state of the Al0.5CrFeCoNiCu alloy. With the aid of high resolution electron microscope, authors identified L12 precipitates in both the dendritic and interdendritic regions of sample aged at 1000 °C for 1000 h. Fine-scale precipitation led authors to suggest that the precipitation characteristics in HEAs are similar to those of conventional alloys. Daoud et al. [25] studied HESA with increased Ni content viz. Al10Co25Cr8Fe15Ni36Ti6 aged at 900 °C for 5–50 h. The alloy showed the relatively high-volume fraction of γ’ phase viz. 46%, with an average size of nearly 450 nm. In terms of properties, the studied alloy showed a high-temperature tensile strength up to 800 °C and a high elongation to failure. For all studied temperature ranges, the alloy’s tensile strength exceeded that of commercial alloys such as Inconel 617 and Alloy 800H. He et al. [8] changed the design concept by specifically inducing the formation of L12-coherent nano-sized precipitates through minor additions of Ti and Al to a single phase (FCC-based) FeCoNiCr alloy. Such a design enhanced the strength without compromising the tensile ductility. The exceptional yield strength possessed by this alloy was attributed to the additive contribution of precipitation hardening, dislocation hardening and grain boundary hardening. Tsao et al. [51] designed Ni-Co-Fe systems of medium and high entropy superalloys and investigated the strengthening due to the L12 γ’ precipitate. They showed that the thermal stability of the γ’ phase can be enhanced by substituting Ni with Ti, which tends to improve the ordering of the γ’ phase. They noted that in comparison to superalloys, HESAs exhibit stable γ-γ’ microstructures without forming TCP phases after exposure at 900 °C for 300 h. Wang et al. [26] investigated the strengthening mechanism of L12 nanoprecipitates on FCC-based Al0.2CrFeCoNi2Cu0.2 alloys. They found that dislocation shearing of L12 nanoprecipitate is responsible for precipitation-hardening of the alloy. The Ming et al. [24] study induced a coarse-grained Al0.2Co1.5CrFeNi1.5Ti0.3 superalloy with nano-sized and coherent precipitates to impart a combination of strength and ductility. The alloy was processed by hot rolling, annealing at 1150 °C for 3 h and thereafter aging at 800 °C. The alloy contains FCC solid solution phase (with an average grain size > 1 µm) and ordered spherical L12 precipitates. Their findings indicate that the average diameter of precipitate increases from 6 nm for 1 h aging to 50 nm for 100 h aging. The yield strength reached a low value of 540 MPa, an ultimate tensile strength value of 917 MPa and an elongation to fracture value of 50%. Zhang et al. [32] designed an Ni45-x(FeCoCr)40(AlTi)15Hfx high-entropy superalloy with excellent γ/γ’ structure. They found that at x = 0.2 and the yield strength reached 1004 MPa at 750 °C, which is comparable to cast Ni-based superalloys. This was attributed to well-distributed γ’ particles that function to prevent dislocation motion at elevated temperatures. Zhao et al. [52] studied the coarsening rate of L12 precipitates of (NiCoFeCr)94Ti2Al4 at elevated temperatures (750–825 °C). The precipitate coarsening rate was therefore found to be one to two orders of magnitude lower than traditional Ni-based alloys. Thus, it implied good thermal stability of the L12 precipitates. Given the domination of the Ni in γ’ phase, subsequent studies designed HESAs with high Ni contents to induce optimum L12 precipitate properties for effective strengthening. Kang et al. [53] synthesized a novel Ni-rich Ni46Co22Al12Cr8Fe8Ti3Mo1 HESA via a powder metallurgical process to achieve a γ’-precipitate-strengthened microstructure. The grain size of FCC matrix, volume fraction of γ’ and size of γ’ were found to be 566 nm, 40.1% and 267 nm, respectively. The alloy showed a high tensile yield strength of 1355 MPa and ductility of 8.7%. These properties were attributed to the grain refinement effect, homogeneous distributed γ’ precipitates, and TiC and Al_2_O_3_ dispersoids. Shafiee et al. [54] developed a wrought high nickel content of Ni46.4Al5Co5Cr21.2Fe15Ti1.5Nb3.1Mo2.8 HESA to study its precipitation behavior. The alloy exhibited a high strength of 1310 MPa and a relatively high ductility of 32% after aging. In comparison to IN718, the HESA in this study shows a superior combination of properties. Zheng et al. [27] designed a CoCrFeNi(Ni_3_Al)x (x = 0.25, 0.5, 0.75, 1) HEA system in order to induce precipitation strengthening through nano-sized L12 precipitates in the FCC matrix. The alloy showed a good combination of comprehensive strength and ductility when x = 0.75. Tensile strength reached 1200 MPa; yield strength reached 910 MPa and elongation reached 14%. Through realisation of the importance of γ’ precipitate size and volume fraction to effective precipitation strengthening, most studies attempted to optimise its content and thus properties by tuning composition and controlled processing. Thus, this further corroborates the importance of relating the microstructure to mechanical properties at all temperatures.

## 3. Strengthening Mechanisms in High Entropy Superalloys

It has been known from the concept of conventional alloys that the strength of the alloy is greater when the dislocation mobility is retarded. With the introduction of a higher complexity in the structure of alloys, strength further increases because of the rugged surfaces the dislocations have to bypass. Thus, HESAs which are known to possess such complexity will inevitably provide the highest strength, unprecedented since the advent of traditional alloys. This is because the strengthening mechanism wherein dislocation motion is impeded to impact the strength of the alloy in traditional alloys also occur in HESAs, albeit in an uncharted fashion due to unique characteristics of HESAs as compared to traditional alloys. However, the basics of such mechanisms in HESAs need to be fully understood and modelled to facilitate the design of these alloys towards exceptional performance. There are six known strengthening mechanisms in alloys which act independently of each other: solid solution strengthening, precipitation strengthening, grain boundary strengthening, work-hardening, transformation hardening and dispersion strengthening. However, in this paper, consideration is given to solid solution strengthening, precipitation strengthening, dispersion strengthening and grain boundary strengthening.

### 3.1. Solid Solution Strengthening

Solid solution strengthening occurs due to changes in lattice parameters caused by solute addition, and the relative valence of the solute and solvent [55]. The catch in HEA/HESA is that there are no definite criteria which distinguish a solute from a solvent. As a result, it is difficult to quantify solid solution strengthening in HESA with a high accuracy. However, Varvenne et al. [56,57] assumed each element in an alloy to be a solute embedded in the average effective medium matrix of the surrounding material. The authors noted that this approximation is effective and well established in various contexts, viz., Electronic Structure Theory (with Virtual Crystal and Coherent Potential Approximation) and Embedded Atom Method Potentials. He et al. [8] based their calculations of solid solution strengthening on a standard model also using the same approximation of effective matrix by treating FeCoNiCr as the solvent matrix and Ti + Al as solutes. Toda-Caraballo and Rivera-Díaz-Del-Castillo [58] noted that there is no reference atom with a lattice that is changed by the presence of solute atoms in HEAs, but there is a variation of the interatomic distance in the crystal lattice around its mean unit cell parameter. Thus, the authors used this approach to model solid solution hardening in HEAs by defining variable unit cell parameters and atomic size misfits of elements. The modeling fundamentals work best for FCC HEAs. In BCC HEAs/HESAs, the general accepted mode of deformation mechanism is kink-pair nucleation for screw dislocation motion. It is important to know how solutes affect the double-kink nucleation process [59]. However, the effects of solutes on this process can soften and/or strengthen the dislocation motion. Thus, the strengthening mechanism in BCC HEAs/HESAs requires a further study in identifying the relevant operating mechanism and development of a related mechanistic theory which deals with arbitrary composition [60]. Therefore, this study excludes modelling of BCC HEAs.

Solute atoms can be categorized into two types according to their strengthening fashion: interstitial atoms, which do not cause spherical distortions and impose relative strengthening effect of approximately 3 × G (G-Shear Modulus) per unit concentration; and substitutional atoms which cause spherical distortion and impose a relative strengthening effect approximately G/10 per unit of concentration [55]. There are six mechanisms identified in the book by Dieter [55] in which solute atoms interact with dislocations in binary systems. Viz. elastic interaction, modulus interaction, stacking-fault interaction, electrical interaction, short-range order interaction and long-range order interaction. Among these interactions, elastic modulus and long-range order interactions are relatively insensitive to temperature because they act at long ranges, and as a result their effects persist until about 0.6 × melting point of the alloy, while the other interactions contribute strongly to the flow stress at lower temperatures because they act at short ranges [55]. Varvenne et al. [57] explained in their study that the model which encompasses all interactions of solute-dislocation do not actually yield an understanding of the role played by average matrix or solute properties, nor dislocation structure, and thus full solute–dislocation interaction energies may not be accurately assessed in real materials. Elastic interaction is mostly considered to model solute–dislocation interactions in alloys.

The standard model for solid solution strengthening is based on how strongly the dislocation interaction is with individual solute atoms on the gliding plane. With an increase in stress or temperature, the pinned dislocation bows out in regions between the solute atom, giving rise to the square-root-power dependence of the strengthening effect on the solute concentration [61,62]. He et al. [63] used the same model for a substitutional atom solution based on the mechanism of elastic interactions to evaluate solid solution strengthening caused by Al + Ti solute in FCC-based FeCoNiCr matrix. Consider the equation below.

(1) $$\Delta \sigma_{s}=M\cdot \frac{G\cdot \varepsilon_{s}{}^{3/2}\cdot c^{1/2}}{700}$$

where M is the Taylor factor (=3.06, a factor that converts shear stress to normal stress for an FCC polycrystalline matrix), G is the shear modulus (the ratio of shear stress and shear strain, measured in GPa), c is the total molar ratio of solutes (Al + Ti) in simple FCC material and εs is the interaction parameter that combines the effects of elastic and atomic size mismatches, i.e., ε_G_ and ε_a_, respectively. Interaction parameter εs is calculated as follows.

(2) $$\varepsilon_{s}=\left|\frac{\varepsilon_{G}}{1+0.5\varepsilon_{G}}-3\varepsilon_{a}\right|$$

(3) $$\varepsilon_{G}=\frac{1}{G}\frac{\partial G}{\partial c};{\;\varepsilon }_{a}=\frac{1}{a}\frac{\partial a}{\partial c}$$

where a is a lattice constant of the FeCoNiCr base alloy matrix. Parameter εa is obtained from refined XRD patterns, thus rendering solid solution stress to be readily estimated. The authors found Δσs for the sample labeled P1 to be 25.4 MPa and P2 to be 14.4 MPa (i.e., samples have the same composition but underwent different processing). With these values, they concluded that solid solution strengthening is insignificant to account for the experimentally determined strength, and thus ascribed the strength to other strengthening mechanisms. Li et al. [64] used the similar model to account a solid solution strengthening induced by carbon addition in the matrix of CoCrFeMnNi HEA. Solid solution of carbon is no more than 1 at.% in CoCrFeMnNi HEAs, and thus the contribution to yield stress was estimated as 13 MPa. Basu and Hosson [65] stated that Friedel’s model of solute solution strengthening is neither applicable in dilute nor concentrated HEAs, as evident from spin-lattice relaxation data and data relayed by strain-rate change experiment on the several alloy system.

**Figure 2:**
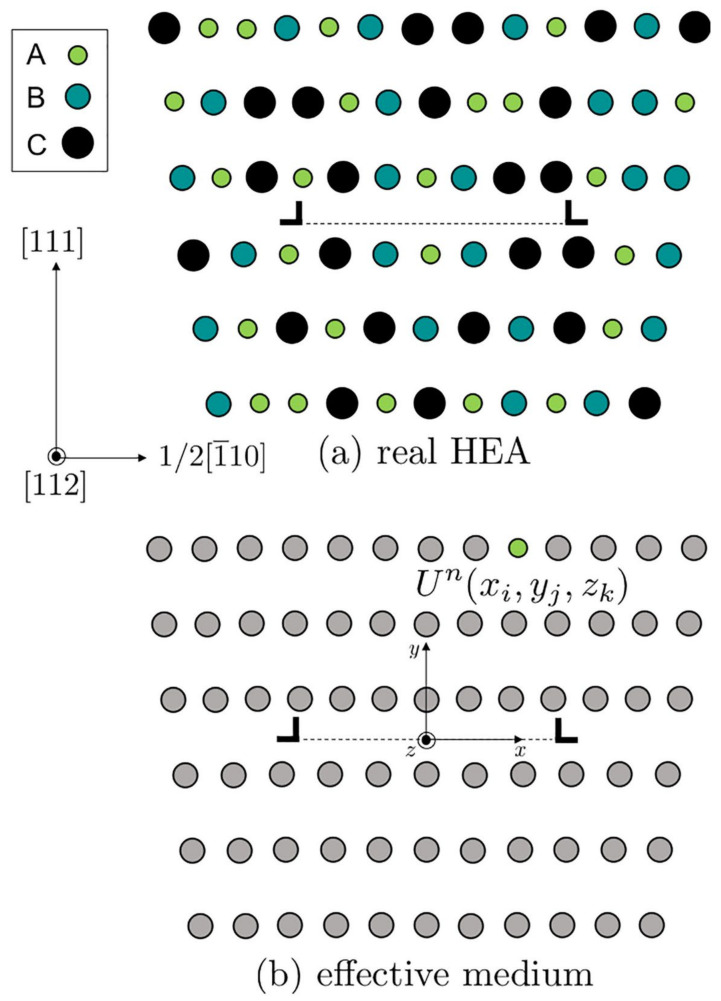
Schematic showing effective medium approach for dislocation/solute interactions. (**a**), Fully-random 3-component (A,B & C) HEA containing a dissociated edge dislocation; (**b**), Effective matrix material of the same alloy, with an embedded A “solute” at position (xi, yj, zk) relative to the dislocation centered at the origin, with interaction energy UA (xi, yj, zk). Adapted from: [56].

Varvenne et al. [56] showed that solid solution strengthening in FCC HEAs can be modeled based on first-principle-computed interaction energies. The contributions to the interaction energy stems from the interaction of the dislocation’s stress field with the misfit strain tensor of the solute and from the chemical misfit resulting from change in the bonding environment of the solutes in partial dislocation core geometry and stacking fault region between them [60]. Figure 2 provides the schematic of [56] concept.

**Figure 3:**
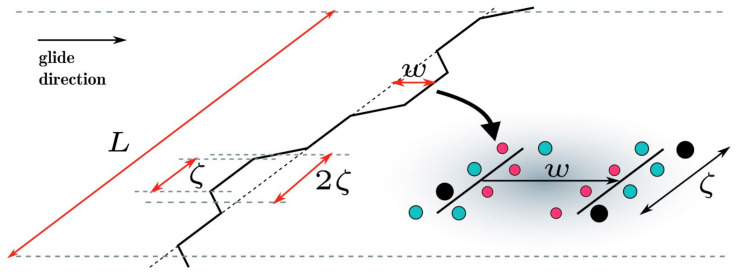
Schematic diagram showing low-energy arrangement of the dislocation gliding over random field of solutes. Adapted from [56].

In this model, solute atoms act as local fluctuations relative to an effective-medium reference matrix that contains dislocations. Due to these chemical fluctuations, dislocation adopts a wavy configuration, characterized by a wavelength 2ζ and amplitude w_c_, in order to find energetically favorable regions, but it does so at a cost of an elastic energy due to the dislocation line tension Γ. The bow-out (ζ,w_c_) property of the pinned dislocation is due to an attempt to balance these two energetic contributions. Thus, through a thermally activated process and applied resolved shear stress, dislocation can unpin and escape from this local energy state [66]. The schematic in Figure 3 shows the concept.

Therefore, based on the mechanism aforementioned and by considering only elasticity contributions to the solute–dislocation interaction of substitutional solid solution (FCC based), the zero-temperature flow stress and energy barrier are calculated as follows, Ref [56] provides a description of its derivative.

(4) $$\tau_{y_{0}}=0.051\alpha^{{-}_{3}^{1}}\mu {\left(\frac{1+\nu }{1-\nu }\right)}^{\frac{4}{3}}f_{1}\left(w_{c}\right)\times {\left[\frac{\sum_{n}C_{n}\left({\bar{\Delta V_{n}}}^{-2}+\sigma_{\Delta V_{n}}{}^{2}\right)}{b^{6}}\right]}^{\frac{2}{3}}$$

(5) $$E_{b}=0.274\alpha^{\frac{1}{3}}{\mu b}^{3}{\left(\frac{1+\nu }{1-\nu }\right)}^{\frac{2}{3}}f_{2}\left(w_{c}\right)\times {\left[\frac{\sum_{n}C_{n}\left({\bar{\Delta V_{n}}}^{-2}+\sigma_{\Delta V_{n}}{}^{2}\right)}{b^{6}}\right]}^{\frac{1}{3}}$$

where α is a line tension parameter (α = 0.123, is obtained from the atomistically measured edge dislocation line tension using embedded-atom method (EAM) in FeNiCr effective matrix), µ is the isotropic shear modulus, b is the magnitude of Burger’s vector, w_c_ is a characteristic distance along the glide plane, ν is the Poisson’s ratio, Cn is the concentration of the solute, $\bar{\Delta Vn}$ is an average misfit volume of the solute, σ_ΔVn_ is the standard deviation of ΔVn due to the local chemical and structural environment. ΔVn of each solute element is calculated using literature data on the lattice parameter of binary Ni-X (X = Co, Cr and Fe) for fcc solid solution system and the Ni-Co-Fe-Cr-Mn HEAs family. $f_{1}\left(w_{c}\right)={\left[{\left(\frac{b}{w_{c}}\right)}^{\frac{5}{2}}\times \underset{\mathrm{ij}}{\sum }\Delta f_{\mathrm{ij}}{}^{2}(w_{c})\right]}^{\frac{2}{3}}$ And $f_{2}\left(w_{c}\right)={\left[{\left(\frac{w_{c}}{b}\right)}^{2}\times \underset{\mathrm{ij}}{\sum }\Delta f_{\mathrm{ij}}{}^{2}(w_{c})\right]}^{\frac{1}{3}}$ are minimized coefficients for the given core structure, their values are $f_{1}\left(w_{c}\right)\sim 0.35\;$and $f_{2}\left(w_{c}\right)\sim 5.7$ for the low temperature solution (~700 K) and they include a wide range of core structures with stacking fault separation above ≈10b (where b is the magnitude of the Burgers vector), which is typical for HEAs [66]. The polycrystalline elastic constant (μ and ν) could be estimated using a rule-of-mixture based on elemental elastic constants, readily obtained from the literature data.

The general model accounting for all mechanisms in which solute atoms interact with dislocations is calculated as follows [55,56], based on description of energy barrier (ΔEb) and zero-temperature yield stress (τ_yo_).

(6) $$\Delta E_{b}=1.22{\left(\frac{w_{c}{}^{2}\Gamma {\tilde{\Delta \mathrm{Ep}}}^{2}\left(w_{c}\right)}{b}\right)}^{\frac{1}{3}}$$

(7) $$\tau_{\mathrm{yo}}=1.01{\left(\frac{{\tilde{\Delta \mathrm{Ep}}}^{4}\left(w_{c}\right)}{{\Gamma b}^{5}w_{c}{}^{5}}\right)}^{\frac{1}{3}}$$

where w_c_ is the spatial range of interaction of the solute with dislocation, ΔẼp(w_c_) is the change in energy (per unit length) of a straight segment of dislocation as it moves a distance w through the solute field. b is the magnitude of the burgers vector, $\dot{\varepsilon_{0}}$ is the reference strain-rate, Γ is the dislocation line tension in the effective matrix ($={\alpha \mu b}^{2}$, where the variables are described above). However, as stated above, this model suffers from shortcomings, and thus it does not provide an accurate description of material behaviour.

The finite-temperature and finite-strain-rate yield stress [τ_y_(T,έ)], by modifying the analysis to logarithmic form to fit full multiscale dislocation bow out over stresses $0.2\leq \tau_{y}/\tau_{0}\leq 0.5$ or higher temperature regimes, is modelled as follows [55,67]:

(8) $$\tau_{y}\left(T,\dot{\varepsilon }\right)=\tau_{\mathrm{yo}}\exp({-}_{0.57}^{1}\frac{\mathrm{kT}}{\Delta \mathrm{Eb}}\ln\frac{{\dot{\varepsilon }}_{0}}{\dot{\varepsilon }})$$

where k is the Boltzman constant, $\dot{\varepsilon_{0}}$ is a reference strain rate (nominally related to dislocation density ρ, burger’s vector b, typical dislocation slip distance d_s_, and attempt frequency v_0_ by relation: $\dot{\varepsilon_{0}}={\rho \mathrm{bd}}_{s}v_{0}$; its precise value is not important thus is set as $10^{4}s^{-1}$ consistent with literature), T is the temperature and $\dot{\varepsilon }$ is the plastic strain-rate. This temperature-dependent solid solution strengthening theory is implicit, because it requires information about misfit volumes of all elements in the alloy at that composition and elastic constants at relevant temperature. 

The authors [55,56,65] stated that the above equations indicate that high strength materials can be achieved by maximising the shear elastic modulus of the matrix and maximising the concentration-weighted mean-squared misfit volume quantity. The concentration-weighted mean-squared misfit volume is related to the lattice misfit parameter δ, which can be estimated using the misfit volume of the solutes computable by ab initio calculations. It is important to note that the number of components and/or equiatomic composition does not lead to high strengthening. The drawback of the elasticity model as represented above is that a chemical-specific core interaction is absent and fluctuations due to local structural and chemical disorder in HEAs are neglected.

**Figure 4:**
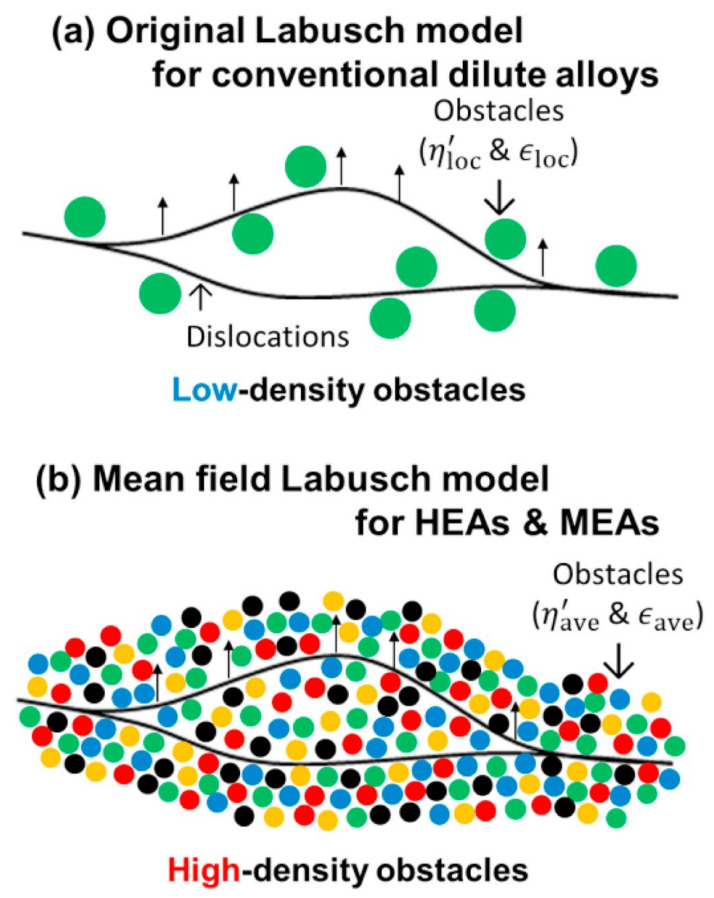
Schematic showing mean field of atoms around dislocation on a slip plane; (**a**) dilute system of typical traditional alloys; (**b**) concentrated system of typical HEAs and MEAs. Adapted from [69].

Toda-Caraballo and Rivera-Díaz-Del-Castillo [58,68] adopted the approach proposed by Gypen and Deruyttere, from the Labush method, to calculate solid solution hardening in HEAs. The model stems from the fact that the presence of solute elements produces a continuous distortion of the crystal lattice, and that the elastic interaction due to atomic size misfit is variable (Figure 4 provide the visualisation). There is no reference atom with a lattice that is changed by the presence of solute atoms; however, the interatomic distance in the crystal lattice varies around its mean unit cell parameter. Therefore, a description of this variable unit cell parameter and the atomic size misfit by the elements characterize the modelling of the solid solution hardening effect in HEAs, see Ref [58] for full description of the model:

The Labusch model is rooted in that the dislocation experiences a pinning effect due to constant interaction with favourable statistical fluctuations of solute-atoms’ fields at higher concentrations (Figure 4b). Using the methodology that is consistent with multicomponent alloys, the model takes the following form [58]:

(9) $$\sigma_{\mathrm{ss}}={\left(\underset{i}{\sum }B_{i}{}^{\frac{3}{2}}X_{i}\right)}^{\frac{2}{3}}$$

where σ_ss_ is the solid solution stress, Xi is the content of solute i and Bi is the constant depended on shear modulus μ of the alloy; the mismatch parameter is ϵi and the fitting constant is Z. Considering the effect of temperature on solid solution hardening, the function of Bi takes the following form:

(10) $$B_{i}=3\mu \epsilon_{i}{}^{\frac{4}{3}}Z_{0}e^{-{\mathrm{mk}}_{b}T/W_{o}}$$

where Zo is the constant that is dependent on the solvent but independent of temperature (experimentally fitted as Z = 5 in Toda-Caraballo’s [68] work), K_b_ is the Boltzmann constant, m = 25 ± 2.3 is a constant, T is the temperature, W_o_ is the constant dependent on the material (describes the binding energy of an edge-dislocation segment with solute atoms in the proximity and is calculated using the following relation).

(11) $$W_{0}=N\sqrt{{U\mu b}^{3}}$$

where b is the magnitude of the burgers vector, U is the Peierls energy per interatomic spacing along the screw dislocation, N is the parameter ranging from 4 to 6 for fcc or hcp (for concentrated solid solutions and low intrinsic lattice friction metals) and from 1 to 2 for bcc (for high intrinsic friction metals), the shear modulus of the alloy µ is obtained from a simple mixing rule of the elemental shear modulus. 

Mismatch parameter ϵi accounts for modulus misfit ηi and elastic misfit δi by the following relation:

(12) $$\in_{i}={\left({\eta^{\prime }}_{i}{}^{2}+\alpha^{2}\delta_{i}{}^{2}\right)}^{\frac{1}{2}}$$

where α is a parameter that accounts for the difference in interaction forces between screw and edge dislocations and the solute atom; 3 < α < 16 for screw dislocations and α > 16 for edge dislocations, modulus misfit η’i and elastic misfit δi can be calculated by the formulas below:

(13) $${\eta^{\prime }}_{i}=1-\frac{\sum_{i}^{n}X_{i}\mu_{i}}{\left|\mu_{i}\right|}$$

(14) $$\delta_{i}=\frac{\delta s}{{\delta X}_{i}}\frac{1}{s}$$

where $\frac{\delta s}{{\delta X}_{i}}$ is the parameter that describes variation of the interatomic spacing with composition and s is the interatomic spacing; $\frac{\delta s}{{\delta X}_{i}}$ is modelled by the following function:

(15) $$\frac{\delta s}{{\delta X}_{i}}=s_{\mathrm{ii}}-X_{k\neq i}S_{k}{X^{\prime }}_{k\neq i}\frac{1}{{\bar{X}}_{k\neq i}{}^{2}}$$

where s_ii_ is the solvent-solvent interatomic spacing, S_k_ denotes submatrix with indices {k = 1,…,n where k≠i}, and X_k_≠i is a vector with the elemental content of the original alloy (where element i has been removed). Toda-Caraballo and Rivera-Díaz-Del-Castillo [58] noted that the hardening effect of each element is linearly proportional to its elastic misfit δi ($\frac{\delta s}{{\delta X}_{i}}\frac{1}{s}$). The author showed that addition of Cr on ternary CoFeNi lead to hardening effect, it was explained by the area of a rectangle defined by corners (0; 0) and (Xi;$\frac{\delta s}{{\delta X}_{i}}\frac{1}{s}$) on a graph of $\frac{\delta s}{{\delta X}_{i}}\frac{1}{s}$ against elemental content Xi. The sum of areas of Co, Fe, Ni and Cr with contents ¼ each gives 8.4 × 10^−6^, while without Cr is 6.9 × 10^−6^, hence an increase in hardening with Cr addition. In the other instance, an addition of Fe on CoCrNi softens the alloy because of the decrement of the sum of areas, viz., CoCrNi is 9.92 × 10^−6^ while CoCrFeNi is 8.4 × 10^−6^. Thus, element addition on the alloy leads to an increased hardening effect if it increases the value of the sum of areas on a graph of elastic misfits against the elemental content. The model proposed shows predictions of yield strength that are in good agreement with results collected from the literature on HEAs.

The predictive model for solid solution strengthening in HEA/HESA is based on approximations. The computation of dislocation core structure and solute/dislocation interaction energy in HEAs is challenging due to the high number of components and the presence of chemical, structural, and magnetic disorder. The models developed thus far assume the effective matrix material and first-order local fluctuations around the average. Due to limitation of ab initio methods on this type of alloy, techniques such as virtual crystal approximation and coherent potential approximation, and special quasi random structures, have been reliably applied to determine the effective matrix properties and chemical disorder [56]. The former techniques require a large number, or at least several simulations, of the average responses of the alloy, whereas local fluctuations tend to cause a deviation from the average to be valid statistically [60]. The latter technique efficiently produces a perfect simulation of disordered state but exclude local lattice distortion [60]. Okamoto et al. [70] used mean-square atomic displacements (MSADs), determined by first-principle total energy calculations, based on a special quasi random structure for the quinary HEA, as a scaling factor of solid solution strengthening in equiatomic CrMnFeCoNi HEA and its quaternary and ternary equiatomic derivatives. They showed that 0 K yield stress is proportional to the square root of average MSADs values. Thus, it corroborates the impact of lattice distortion on the strengthening of alloys, whereof the lattice distortion has been assumed to be intrinsic in HESAs.

**Figure 5:**
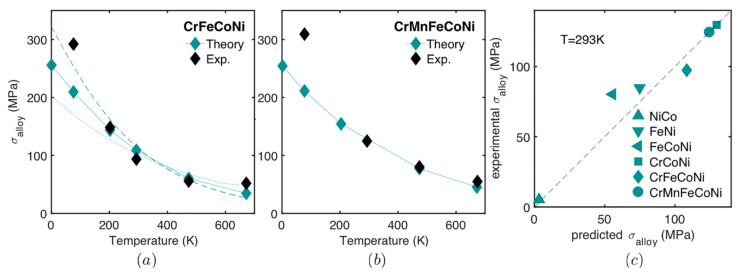
Comparison of experimental and theoretical values in terms of yield strength as a function of temperature (**a**) of NiCoFeCr and (**b**) FeNiCoCrMn equiatomic HEAs. (**c**) Experimental yield strength as a function of predicted yield strength for Ni-Co-Fe-Cr-Mn family at T = 293 K. Hall-Petch contribution to strength has been subtracted. Adapted from [56].

**Figure 6:**
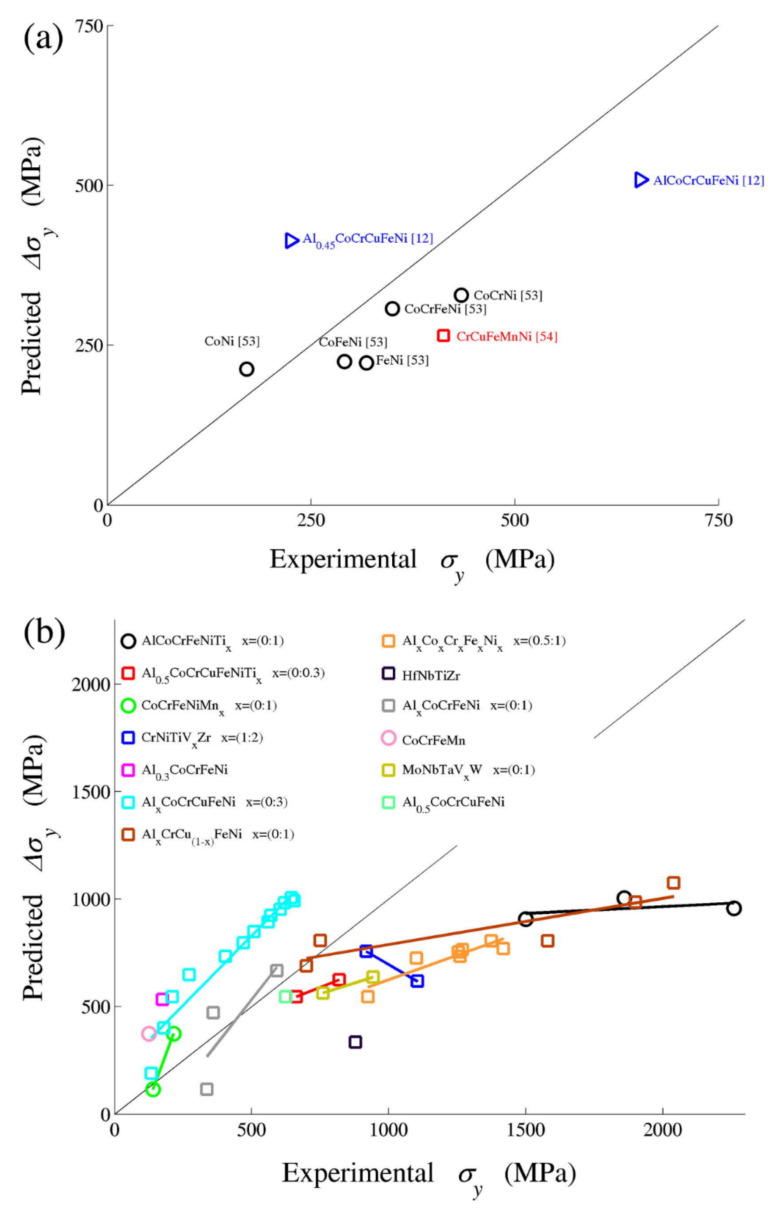
Predicted yield strength as a function of experimental yield strength from solid solution strengthening. Adapted from [58].

**Figure 7:**
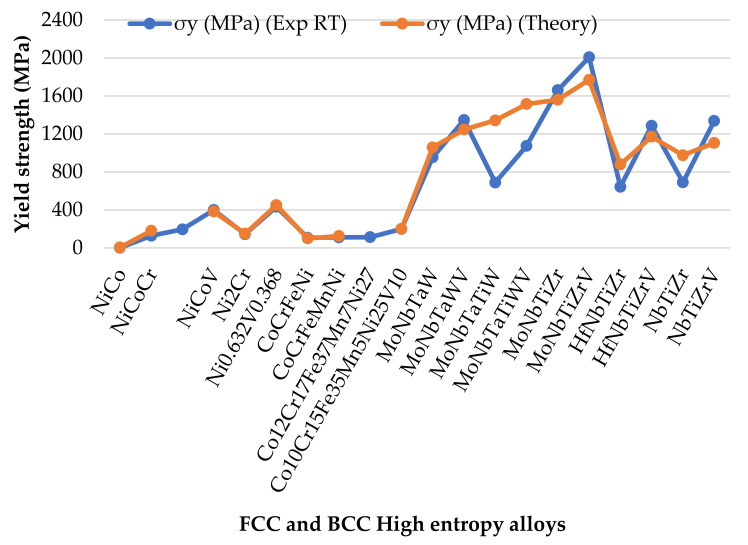
Yield strength (σ_y_) against types of HEAs/HESAs where experimental value at room temperature (RT) is compared with theoretical value (determined by Varvenne et al. [56] model). Data adapted from [74].

All models thus far assume a random solid solution, which is not applicable in HEAs owing to the enthalpy-driven phase reordering during thermomechanical processing in most HEA microstructures [65]. The theories of these models neglect factors such as specific solute–solute interactions and short-range order, applicable even in random alloys [71]. Approaches of solid solution modelling by Varvenne et al. [56] and Toda-caraballo [58] are similar in the sense that they show the direct dependence of the HEAs’ yield stress to shear modulus and the size misfit quantity [60]. Varvernne et al. [56] derivation involves additional material parameters such as dislocation core structure, line tension and standard deviation of average misfit volumes, while Toda-caraballo [58] embed such into an adjustable parameter. Table 1 provides a summarised comparison of solid solution strengthening models. Walbruhl et al. [72] suggested an empirical approach of solid solution strengthening in HEAs that reliably predict yield strength to the accuracy of ±13%. They defined the strengthening parameter A that is optimised to give the best fit with experimental hardness data. For a high temperature effect, the adjustable parameter Q of each element’s binary pairs and structure is defined experimentally. However, no physical meaning can be extracted from this approach, and thus the fundamentals of the material behaviour will not be understood. A plateau in the yield strength has been observed at temperatures higher than 1/3 of the absolute melting temperature of the alloys [56]. This effect was attributed, as proposed by Labusch, to dislocation segments that largely jump backward as much as forward when crossing obstacles under thermal activation. However, a more elaborate explanation is required to provide the clear physical origin of this phenomenon. Solid solution strengthening significantly depends on the displacement of atoms from their ideal position [71]. The addition of elements in an alloy therefore leads to a hardening effect when they increase the average misfit volume of the solutes, pertinent to the elastic interaction model. Figure 5, Figure 6 and Figure 7 show the predictions of the developed models in comparison to experimental data; the graphs and the data have been adopted from the studies mentioned. Despite the several assumptions employed in theories of solid solution strengthening, the predictions are good; however, their assessment and validations are limited to well establish experimental systems, and thus their application to other alloy systems might deviate [72]. The good quality of the models is that there are no fitting or adjustable parameters, and thus the analysis is physically based, and atomic and mesoscale material parameters such as solute volume misfits, dislocation topology and chemical ordering can be inferred, controlled and optimised by composition selection and processing conditions.

**Table 1:** Comparison of solid solution strengthening models in terms of their technical characteristics.

	Standard Model (Friedel)	Varvenne et al. [56] Model	Toda-Caraballo and Rivera-Díaz-Del-Castillo [58] Model
Input Material Properties	Solute concentration, interaction parameters, Burgers vector and shear modulus of the matrix.	Elastic constants, lattice parameters, dislocation core structure, dislocation line tension, accurate elemental misfit volumes in the alloy, at composition of interest.	Lattice parameters, binary interatomic spacing, elastic constants and the dislocation line tension of the average matrix.
Assumptions	Only solutes atoms on the gliding plane interact with dislocation.	Solute do not alter the core geometry of the dislocation.	Solutes do not interact with each other, or their interaction is negligible.
	The alloy is dilute, where a base element makes the host, and other small quantity elements are solutes.	Single phase FCC random alloys—thus neglect possible short range ordering effects and transformations to multiphase materials.	The general interatomic spacing between solutes i and j is independent of concentrations Xi and Xj and atoms around i and j.
		Individual atomic volumes are fixed.	Vegard’s law is applied to approximate the variation of cell parameter in a binary alloy.
		Unique and fixed value per studied alloy for line tension is assumed.	Assume dilute-limit labusch-type analysis.
		The alloy is elastically isotropic for the dislocation pressure field.	Elastic misfit contribution to strengthening.
			Use generalized size and modulus misfit parameters to fit existing data.
Predictions of yield strength values relative to experimental values	The quantitative predictions are elusive [62]	The model prediction of the strength versus temperature and strain rate is very good for alloys NiCoFeCr and NiCoFeCrMn, with no fitting parameters [73]. However, the predictions are below the experiments at lowest temperatures (77 K). For the studied alloys NiCo; NiFe; NiCoFe and NiCoCr at the temperature of 293 K, the predictions are reasonably accurate, similar to those of simpler dilute binary alloys [73].	Agreement is good for limited alloys studied, the observable deviation is attributed to accuracy of elastic misfit and for other interactions, such as stacking faults, valence, short range order and long-range order.
Drawbacks	Since only solutes along the glide plane are considered, the model misses the interaction energies of solutes off the glide plane, which are substantial in the vicinity of the dislocation.	The model does not consider atomic fluctuations at the scale of b < ζ, wc because the line tension concept would be invalid. Although such fluctuations are not calculable, they could generate small additional energy barriers that would contribute to strengthening at zero temperature but are ineffective at finite temperatures.	The computation of unit cell parameters of a HEA shows an overestimate for BCC HEAs and an underestimate for FCC HEAs, and thus a correction factor is involved in the calculations.
	Application of the model at concentrations of the order of 1%, typical of engineering alloys, is questionable [62].	The solute/dislocation interaction energies may not be easily computable in real materials.	
	The model suffer difficulty to describe material with complex chemical structures, i.e., precipitates, mixed FCC plus BCC structure.		
	The model is applicable only when the solute obstacles are strong and have a low concentration.	Line tension effect is not precisely known [60].	
		More accurate and detailed calculations of misfit volume, dislocation core structures and interaction energies with solutes are needed.	
	The models are describing the solid solution strengthening for substitutional elements but do not attempt to include the distinctive interstitial elements.		
	Models do not include a particularly important electronic contribution to solute–dislocation interaction.		

### 3.2. Precipitation and Dispersion Strengthening

The strengthening due to second-phase particle supplements the solid-solution strengthening produced by the matrix [55]. For alloys produced by equilibrium methods, second-phase particles nucleate because of the matrix supersaturation, and thus their production ensures maximum solid solution hardening. In addition to the above contribution to strengthening, the presence of second-phase particles in a continuous matrix induces localized internal stress, which impacts the dislocation mobility in the continuous phase [55]. Strengthening from second-phase particles depends on the following factors as mentioned in the book by Dieter [55]: size, shape, number and distribution of the second-phase particles; the strength, ductility, and strain hardening behaviour of the matrix; and in second-phase, the crystallographic fit between the phases, and the interfacial energy and interfacial bonding between the phases. The author further states that it is challenging to measure these factors with accuracy because of complex interrelations between them. In precipitation hardening, second-phase particles are in solid solution during elevated temperature but precipitate upon quenching and aging at a lower temperature, and as a result, they tend to be coherent with the lattice of the matrix. While in dispersion hardening, second-phase particles have negligible solubility in the matrix even at high temperatures and as a result there is no coherency between second-phase particles and the matrix. Generally, second phases are observed in HESAs/HEAs and conventional theories are applicable to these alloys. However, as elucidated by Yeh et al. [11], the matrix and precipitates made with multi-elements in HEA might be stronger compared to conventional alloys and thus have a higher strength level. 

There are two general mechanisms of precipitation hardening: shearing mechanisms, where precipitate particles are sufficiently small and coherent with the matrix, and dislocation-by-pass mechanisms (Orowan), where the radius of the precipitate particles exceeds a critical value or is incoherent with the matrix. 

**Figure 8:**
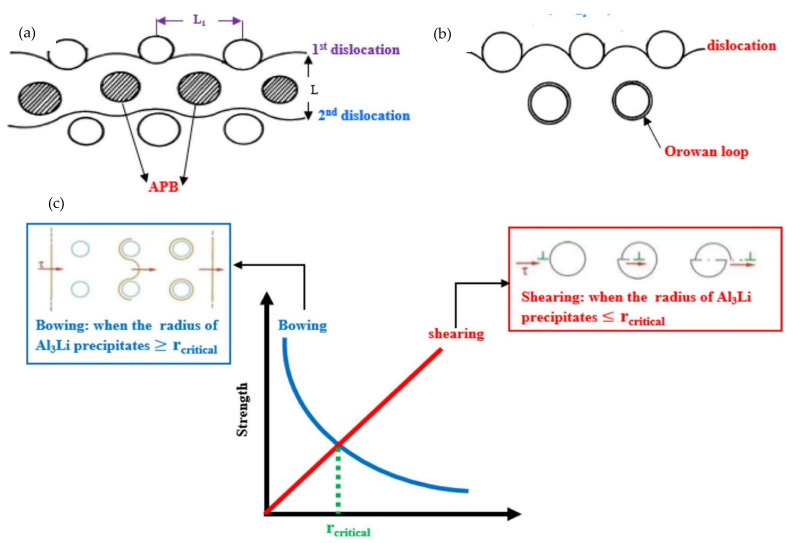
Schematic showing interaction modes between dislocation and precipitate particles (**a**) Shearing mechanisms (**b**) Orowan dislocation-by-pass mechanism (**c**) the comparison between shearing and bowing mechanism in terms of strength as a function of precipitate size. Adapted from [76].

In the modelling of the shearing mechanism, three contributing factors are involved [63] viz. particle-matrix coherency (Δσ_cs_), modulus mismatch (Δσ_ms_) and atomic ordering (Δσ_os_). The former two contribute prior shearing of particles, while the latter contributes during shearing of particles, the criteria being that the larger one, between Δσ_cs_ + Δσ_ms_ and Δσ_os_, determines the resultant contribution, since they occur in sequential process [63]. Δσ_cs_ contribution of shearing mechanism is due to the interaction of dislocation with the coherency strain field in the matrix around the coherent particle [75]. Δσ_ms_ is due to that particle raise or lower the energy of a dislocation passing through them because they have a modulus which is significantly different from the matrix [55]. Δσ_os_ is due to increase in the particle/matrix interfacial energy, where the dislocation passing through a particle leaves in its wake an anti-phase boundary (APB) with an associated disordering energy (Figure 8a) or a stacking fault within a particle with its associated stacking fault energy [75]. These produce a hardening effect, modelled by Equation (in Table 2). In modelling the Orowan mechanism (Equation (16)), consideration is given to the shear stress required to bow a dislocation line between particles separated by distance ℓ (Figure 8b) and considering the effects of statistically distributed particles.

(16) $$\sigma_{\mathrm{Or}}=\frac{\mathrm{Gb}}{2\pi l}\ln\frac{l}{r_{0}}$$

where M = 3.06 (Taylor factor for FCC matrix), f-volume fraction of the precipitates, r-radius of a spherical precipitate, α_ε_ = 2.6 for FCC structure, m = 0.85, ε ≈ ⅔∙(Δa⁄a)-constrained lattice parameter mismatch where Δa is the difference of lattice constant between Ni3(TiAl) phase and the FCC matrix calculated by XRD results, ΔG is the shear modulus mismatch between precipitates and matrix, l-average edge-to-edge inter-precipitate distance, r_0_-dislocation core radius (r_0_ ≈ b) [24], b = 0.262 nm (Burger’s vector of ½ < 110 > dislocations in Ni3Al), G = 78.5 MPa (shear modulus), and γ_APB_ = 184 mJ/m^2^ (anti-phase boundary energy of binary L12 Ni3Al). Ming et al. [24] studied the nature of the dislocation interaction with the nanoprecipitates to demonstrate the precipitation hardening mechanism in Al0.2Co1.5CrFeNi1.5Ti0.3 HESA. They showed that coherent and smaller precipitates prevalent in samples aged 1–5 h causes gliding dislocations to cut through the precipitates, giving rise to precipitate shearing stress (σ_Sh_) as calculated by Equation in Table 2. Increased size of precipitates that are non-coherent due to over-aging causes gliding dislocations to bypass precipitates by looping and is calculated by Orowan dislocation looping stress (σ_Or_) given in Equation (16). The authors further noted that dislocation cutting mechanisms generate a residual defect with a small Burger’s vector around the precipitate due to lattice mismatch, which leads to smaller back-stresses and results in a lesser strain hardening effect. Dislocation loops created around the precipitates due to Orowan dislocation mechanisms have a relatively larger Burger’s vector, leading to higher back-stresses and thus high strain hardening effect.

The strength of the alloy and the transition from one mechanism to another depends on the characteristics of the precipitation particle. The strengthening of soft particles increases with the particle size; however, the opposite is true with hard particles because of increased particle spacing. Therefore, there is a critical particle size that gives maximum strengthening, and the magnitude of that maximum depends on the magnitude of the fractional coherency strain (particle-matrix) and the volume fraction of particles [75]. The transition from shearing mechanism to Orowan looping mechanism is connected to particle critical size (Figure 8c), whereby the critical size decreases with the particle stiffness [75]. As elucidated by strain hardening effect, the large precipitation particles result in relative high strengthening. Like Ni-based superalloys, high entropy superalloys (HESA) precipitation strengthening is rooted in the presence secondary particles (γ’ phases). Dislocation motions are thus affected by the lattice misfit and coherency at the γ/γ’ interface. As mentioned by Shafiee et al. [54], lattice misfit (δ) is determined by the equation: $=\frac{2\left(a_{\overset{’}{\gamma }}-a_{\gamma }\right)}{a_{\overset{’}{\gamma }}+a_{\gamma }}$, where aγ’ and aγ are the γ’(precipitation phase) and γ(matrix phase) lattice parameters respectively. Yim et al. [16] calculated dispersion strengthening of TiC on CoCrFeMnNi HEA using the equation above, derived from the Orowan dislocation bypassing mechanism. The dispersion strengthening contribution was estimated as ~170 MPa; considering the overall contribution from various strengthening mechanisms the yield strength amounted to 631 MPa, which is quite close to experimental value of 698 MPa, with the difference of 67 MPa attributed to strengthening by oxide particles which was not accounted for. Other studies have also used Orowan dislocation bypassing mechanism to theoretically determine precipitate strengthening due to carbides, i.e., Wang et al. [13] in which the precipitate phases viz. (V,Cr)C3 and V2C assumed different crystal structure and were incoherent with the FCC matrix; the calculated contribution is 90MPa; also showing reasonable agreement with experimental yield strength wherein the overall calculated yield strength was 1052 MPa compared to experimental value of 1045 MPa.

Tsao et al. [23,77,78] elucidate that the strengthening of Ni-based superalloy relies primarily on the growing additions of γ partitioning elements such as Mo, W, Re and Ru. Nonetheless, in HESAs, the γ’ precipitation strengthening is relatively stronger due to the γ’ phases being more highly alloyed. In addition, high Ti content in γ’ phase of HESAs enhances the anti-phase boundary, which plays a significant role in strengthening the γ’ phase. Moreover, the lattice distortion strengthening that prevails in the HESA increases the strength of this alloy as compared to the superalloy. However, the γ matrix in HESA is weaker; as a result, it retards the creep performance of HESAs. Another issue is that γ’ tends to coarsen at higher temperatures, thus reducing further the creep resistance of HESAs. A positive misfit between γ and γ’ also leads to a weak restriction against dislocation motion at high temperatures. Taking all these into consideration makes the prospects of HESA second phase strengthening superseding that of superalloys in high temperature conditions less clear. 

### 3.3. Grain Boundary Strengthening

Gao et al. [38] elucidated that grain boundary strengthening arises from the strain hardening of the region near the grain boundary as a result of dislocation pumping from grain boundary ledges under the elastic incompatibility stresses between adjacent grains before macroyielding. Dieter [55] ascribed grain boundary strengthening to mutual interference because of a slip within the grains. The Hall–Petch equation expresses the flow stress dependence on grain-size and this mathematical model is based on the concept that grain boundaries act as barriers to dislocation motion.

(17) $$\sigma_{0}=\sigma_{i}+{\mathrm{kD}}^{{-}_{2}^{1}}$$

where σ_0_-yield strength, σi-friction stress, k-locking parameter and D-grain diameter. Grain size refinement therefore increases the strength of an alloy because it implies a relatively higher volume fraction of grain boundaries, wherein the dislocation motion is impeded.

Sathiyamoorthi et al. [7] used the modified Hall–Petch relation of the alloy CoCrFeNi, which showed two phases (FCC + Cr_7_C_3_) to estimate strengthening from grain boundary and phase boundary. They estimated the overall hardness of 578 HV, which agreed well with the measured hardness of 580 HV. Kong et al. [14] studied the Ni46Co22Al12Cr8Fe8Ti3Mo1 high entropy superalloy, which they fabricated using a powder metallurgy process. Using the above Hall–Petch equation, they estimated the grain-boundary strengthening of 760 MPa on a sample with a least grain size of 506 nm. They found that the calculated strengthening effects approximate the experimentally obtained yield strength, thus proving the rationality of the assumptions proposed by the strengthening models. Yim et al. [16] studied CoCrFeMnNi HEA reinforced with TiC. This composite alloy was fabricated by mechanical milling and spark plasma sintering, and the resultant grain size averaged to 5.1 μm. Using the Hall–Petch equation, the grain-boundary strengthening contribution was estimated to be 218.7 MPa. The overall strengthening of the composite alloy was calculated to be 631 MPa, which is therefore close to the experimental value of 698 MPa.

As noted by Kong et al. [14], the grain boundary strengthening is not effective at elevated temperatures because small grain size tends to be detrimental to deformation resistance at high temperatures. The reason behind this, as explained by the authors, is the prevalent occurrence of grain boundary sliding at high temperatures, which acts as a diffusion path, thus weakening high-temperature creep resistance. The theory of grain boundary strengthening by Hall–Petch is not rigorous with respect to relationships with alloy chemistry [71], and thus the effect of a characteristic concentrated solution in HEAs is insignificant in this regard, unless an indirect effect such as dislocation density at grain boundaries and easy grain refinement during alloy processing are considered.

## 4. The Impact of Processing Route on Alloy Performance

**Figure 9:**
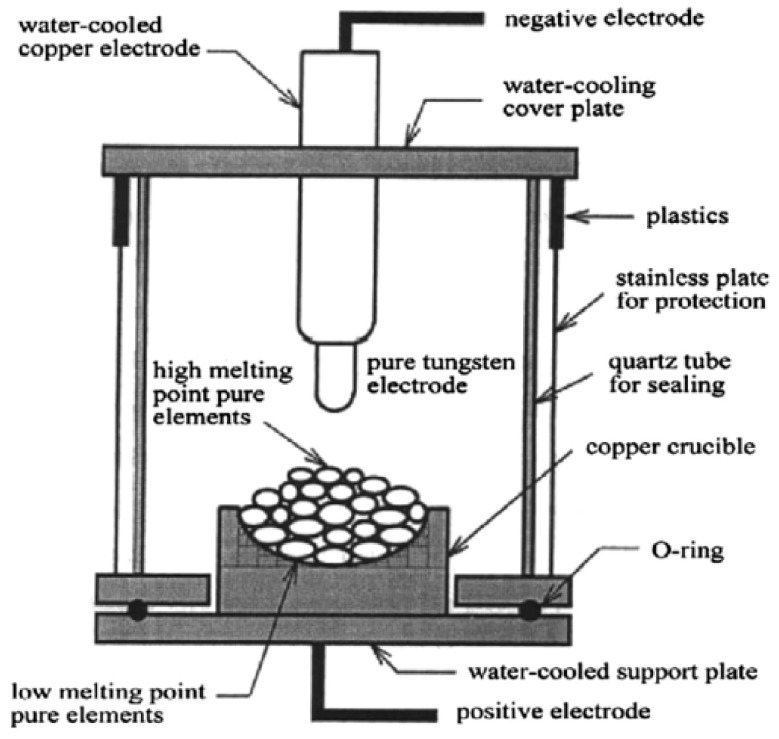
Schematic diagram of the arc melting method. Adapted from [79].

There are three routes of processing techniques viz. liquid-state mixing, solid-state mixing, and gas-state mixing. The most-used processing technique to date in HEAs/HESAs is liquid-state mixing. In liquid-state mixing, constituent elements are fully melted and mixed in a liquid state followed by solidification in a copper crucible. The most-used fabrication method is arc melting; Figure 9 shows the schematic diagram of the operation method. Shortcomings of the arc melting process, as mentioned by Gao et al. [37], include difficulties in controlling the solidification process by virtue of an inevitable rapid solidification, which causes changes in microstructural characteristics in regions near the surface and the centre. Thus, this leads to a limited versatility in fine tuning macroscopic properties. Another shortcoming includes unavoidable casting defects (such as cracks, porosity, elemental segregation, etc.), negligible formation of equilibrium phases, and the presence of residual stresses in the alloy product [32,38]. These significantly degrade the mechanical properties of the as-cast alloy. Therefore, thorough measures need to be implemented when fabricating HEAs/HESAs via this route. 

**Figure 10:**
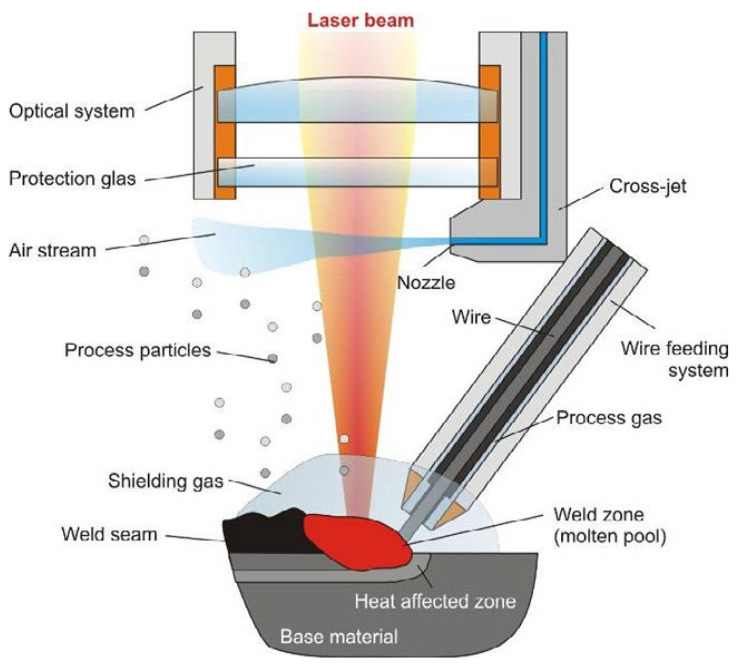
Schematic of laser cladding operation. Image adapted from [81].

Other less-used liquid-state mixing techniques are the Bridgeman-solidification technique and the laser-melting and laser-cladding technique. Gao et al. [38] noted that, compared with the arc melting technique, the Bridgeman-solidification technique provides microstructural control and thus optimised properties of HEAs and this is attributed to the formation of single crystals, made possible by the longitudinal direction of thermal conduction and extraction. On the other hand, the laser-melting and laser-cladding technique utilises the heat from a laser made by a concentrated beam of energy. Such a feature narrows the heat-affected zone, minimises the possibility of cracking, voids, deformation and refines the microstructure. This technique is mostly used to coat the surface of the material for intended properties. The working principle of this technique is shown in the diagram in Figure 10. Another inevitable feature of all the casting processes is that it results in a severe degree of microsegregation. Senkov [80] noted in their study of Al10Nb15Ta5Ti30Zr40 refractory HEA that such elemental microsegragation has a very weak effect on the yield stress of the as-cast alloy; however, it slightly increases the strain-hardening rate after yielding.

The solid-state mixing technique includes mechanical alloying through milling followed by sintering. In this route, mixing of constituent elements is accomplished in solid state via blending of elemental powders. Gao et al. [38] elucidated that the crystalline microstructures produced from milling are very fine grained; hence, this processing route is usually employed for making nanocrystalline materials. The authors further mentioned the advantages of mechanical alloying viz. versatility, compatible to any material including ductile metal alloys; and brittle intermetallic compounds; and composites, applicable to synthesize alloys with very different melting temperatures or vapour pressures. However, the authors noted setbacks such as contamination emanating from milling media or atmospheres. Gao et al. [38] further states that the as-milled powders of HEAs produced by mechanical alloying have a nanocrystalline microstructure. If such a microstructure is maintained after the consolidation process, it may enhance properties such as hardness and strength; however, it may be detrimental to the stability of the phases at a high temperature (due to the high energy possessed by large grain boundary areas in nanocrystalline materials). In vapour-state mixing, only the vapour deposition method is employed to process HEA films in carbides and nitrides. As elucidated by Gao et al. [38], the deposition of the chosen HEA film on the surface of a workpiece is achieved via condensation of a vaporised material. The resultant microstructure of the film depends on parameters such as form of source material, power, base pressure, atmosphere composition, etc. 

The following studies are based on HESAs synthesized by melting and casting, and a comparison thereof with the HESAs synthesized by the powder metallurgy route, and consequently the analysis of the resultant microstructure in relation to processing. Stepanov et al. [6] produced four non-equiatomic HEA alloys of the Fe-(Co, Mn)-Cr-Ni-Al-(Ti) family by arc melting. The XRD data of the as-cast alloys revealed that the Fe36Mn21Cr18Ni15Al10 alloy consists of BCC and B2 as principal phases and a minor FCC phase, the only alloy on the four studied by the authors that show a superalloy microstructure. The disordered BCC matrix phase has a coarse grain size estimated at d ≈ 200 µm with irregular shape, and within them cuboidal precipitates are seen. Higher magnification revealed a discontinuous layer of the FCC phase with a thickness of several microns on the boundaries of these coarse grains. The BCC-B2 interfacial mixture was produced due to decomposition of the high temperature BCC phase formed at the initial stage of solidification, wherein some initial BCC phases were replaced by an FCC phase during cooling. This is corroborated by the microstructure morphology, where FCC grains are separated by thick layers of the BCC-B2 mixture that have fine plates, which indicate sequence of transformation. Shaysultanov et al. [82] studied similar Fe36Mn21Cr18Ni15Al10 alloys fabricated by casting, and showed that in the as-cast condition, the B2 phase is present in a fine, cuboid shape and are uniformly distributed in the matrix BCC phase; however, there was no trace of the minor FCC phase. No study, according to the authors’ records, has attempted to produce this alloy via the solid-state route. A more specific study that provides a comparison between same HESA fabricated by the casting route and powder metallurgy is that by Moravcikova and Gouvea [83]. They observed that the Al0.2Co1.5CrFeNi1.5 alloy in powder metallurgy processing has an FCC solid solution and ordered L12 and BCC phases at the sintering temperature of 1100 °C, which were retained to room temperature due to rapid cooling typical of the sintering process. Nucleation and growth of laves and sigma precipitates were avoided because of short processing times in sintering, wherein effective diffusion is negligible. A cast alloy of Al0.2Co1.5CrFeNi1.5 shows the formation of a complex multi-phase microstructure such as laves, sigma and BCC phases in addition to the FCC solid solution as the dominant phase. Other effects of processing on microstructure and composition distribution detected by the authors are that sintered samples have a fine-grained size (FCC-0.42 µm), while cast alloys show a larger grain size distribution (FCC-42.8 µm and laves phase-5.4 µm), and FCC matrices of both processing have the same composition viz. Fe-, Ni- and Co-rich, the difference being that on cast alloy Ti, the partition is in the FCC matrix phase, while it is depleted in the FCC matrix of sintered alloy because it tends to react with C to form TiC. Fine grain size in sintered alloy is ascribed to a high thermal stability of the TiC particles, which prevent grain growth of the FCC matrix by grain boundary pinning, and a consequence of the spark plasma sintering rapid densification of the severely plastically deformed mechanical alloyed powders. A large grain size of cast alloys show one preferential orientation because of heterogenous nucleation which makes dendrites to crystalise in a specific direction of heat dissipation. Cast alloys therefore are usually followed by additional processing such as homogenization, including hot working or cold working. Powder metallurgy processing has an inherent tendency to form in situ carbides and/or oxides due to process control agents and powders’ exposure to air after milling. Despite the inevitable contamination of HESAs produced by powder metallurgy, the route evidently provides alloys with a mechanical performance superior to that of cast HESAs. Another study which compared casting and powder metallurgy (PM) effects on the performance of HEAs is that by Fu et al. [84]. Although the alloy shows only FCC solid solution phase at room temperature, it is worth mentioning without any regard to the classification of HESA. Fu et al. [84] studied a Co25Ni25Fe25Al7.5Cu17.5 alloy prepared by mechanical alloying and spark plasma sintering (SPS) on the one hand, and vacuum arc-melting on the other hand. Mechanical milling to blend elemental powders was done at 300 rpm for 49 h, followed by consolidation through spark plasma sintering at temperature of 1000 °C with the heating rate of 90 K/min and a constant pressure of 30 MPa. For the casting route, elemental powders were blended for 12 h and pressed at 400 MPa, followed by melting repeatedly at most four times to improve the homogeneity of the ingot. Both the PM and cast HEA exhibit a single solid-solution phase with an FCC crystal structure, and of a similar chemical composition. PM HEA, after sintering, consists of a bimodal microstructure of nanoscale grains (average diameter ~24 nm) possessing irregular morphology and ultra-fine grains (average diameter ~94 nm) in small volume fraction of ~7%. The cast HEA exhibit uniform microstructure with large, elongated grains having average diameter of ~397 μm. Nanoscale twins are observed in the alloy processed by PM. They either stem from deformation twins retained from milled powders or annealing twins created during SPS, as theorised by the authors [84], it implies that planar slips occur during the process. They further noted that strain hardening in the coarse-grained cast alloy is more significant than in the PM alloy, attributed to easy dislocation glide given the presence of sufficient space in cast alloy. The yield strength, fracture strain and hardness of Co25Ni25Fe25Al7.5Cu17.5 HEA prepared by PM is reported as 1795 MPa, 10.6% and 454 Hv, respectively. Evidently possessing a superior combination of mechanical properties as compared to cast HEA viz. Yield strength of 192 MPa, fracture strain of >44.2% and hardness of 129 Hv. One of the first-generation alloys of 3d-transition HESA was studied at both conditions of cast and powder metallurgy by separate authors. Yeh et al. [45] fabricated Co1.5CrFeNi1.5Ti0.5 HEA by arc melting and a Bridgeman solidification technique. After arc melting, the alloy showed as-cast dendrites. The microsegregation was observed on the as-cast samples. Interdendritic regions contained precipitates that XRD analysis revealed as a hexagonal η phase on the study. It is noted that subsequent heat treatment or post-casting processes are a requisite to fine tune the microstructure. Moracvik et al. [85] fabricated a similar alloy, i.e., Co1.5CrFeNi1.5Ti0.5 HEA by a powder metallurgy process viz. mechanical alloying (MA) and spark plasma sintering (SPS). The mechanical milling to mix the powders was carried out at the speed of 250 RPM for 35 h, followed by spark plasma sintering at 1150 °C for 20 min and pressure of 350 MPa to consolidate the mixed powders. The sintered alloy showed a very fine grain size (average = 2.3 μm) because of the synergistic effect of severe plastic deformation of the powder’s material induced by the process of MA and the short sintering time of the SPS process. It was observed that the second phase L12 particle precipitated from the FCC matrix phase upon slow cooling after sintering. In addition, there was the presence of TiO inclusion ascribed to oxygen contamination during the process. It is worth underlining that the microstructures that characterise HESAs, i.e., FCC + L12, are quite prevalent in alloys fabricated by the powder metallurgy route with no second processing, such as annealing and aging. However, to optimise the diameter and volume fraction of the second phase, and to completely avoid detrimental brittle phase, second processing might play an important role, as explained in Section 2. 

HEAs produced by the melting and casting technique show significant shortcomings in the product’s microstructural and macrostructural features as shown by the studies above. It is therefore necessary for this route to be followed by heat treatment or thermomechanical methods to tune the product for the intended properties. However, Ma et al. [86] tell us that the as-cast dendrites induced by wide freezing range of different elements in HESAs and HEAs produce a complex network of dislocations. These might, according to the author, infer the significant grain-boundary strengthening in the form of a pile-up of dislocations at the grain-boundary and by a strain accommodation to maintain strain continuity across grain boundaries. With the milling and sintering technique, many of the disadvantages suffered by casting and melting are eliminated, although the technique has its own shortcomings, such as the cost of materials and the inevitable contamination of the product. In terms of the microstructural features, the solid state route tends to form a nanocrystalline structure, which enhances the properties of the product. Sometimes, subsequent heat treatments or thermomechanical processing are not necessary; however, as noted by Gao et al. [38], the saturated solid solution phases in HEA produced by mechanical alloying may be metastable in the as-milled powders. The formation of precipitates or second phase particles is quite prevalent in the alloy processed by milling and sintering, and thus precipitation strengthening of the alloy can be achieved efficiently. Therefore, in comparison to the casting processes, the milling and sintering process gives better alloy control, and thus it is recommended to fabricate complex system such as HEAs. Many of the HESAs to date have been fabricated by casting and melting. Table 3 indicates the effect of processing on the microstructure and consequently on the mechanical properties of the HESAs identified by the author. 

## 5. The Future of High Entropy Superalloys

The development of a method for integrating characterization techniques will be advantageous to reveal structural features at multiple length scales. As pointed out by [65], strengthening in HEAs might get a contribution first from an atomic scale where local chemical ordering changes the ease of dislocation mobility and dislocation line tension, second from a nanometric scale where ordered cluster formations and nano-sized precipitates produce coherency strain fields that create considerable back stresses to dislocation, thirdly from a median scale where the presence of ordered precipitates creates interphase boundaries and thus order hardening, and lastly from a sub-micron or micrometer scale where defect structures, including grain boundaries, crystallographic dissimilar phase boundaries and twin boundaries influence alloy strengthening through an interaction with line defects. The possibility of HESAs completely surpassing superalloys in structural applications is appreciable because of the immense scope of HEA in general and the potential they have shown so far in terms of an attractive combination of high temperature properties. However, further studies to completely gain a mechanistic understanding will enable a microstructural design to fine-tune the required properties. As noted by Varvenne et al. [57], a high throughput computing of model’s (solid solution strengthening) inputs such as elastic constants, Burgers vector, complex line tension, dislocation core structure and solute/dislocation interactions from reliable ab initio methods and application of the model to predict strength, will enable the creation of design maps for new materials. Walbruihl et al. [72] argued that for a model of solid solution strengthening to find application in a wide system range, especially of HEAs, numerous system-specific parameters need to be assessed, which appears to be infeasible. Thus, they recommended an empirical approach involving a global strengthening parameter optimised to give the best fit with experimental data. Coupled with thermodynamic modeling, which predict alloy phase stabilities, the mechanistic approach can establish a systematic way of identifying composition to achieve high-performance HEAs. The strengthening models developed are thus based on approximations. It is difficult to validate the developed strengthening models by comparison with experimental values because the data from the literature shows considerable scatter and the mechanical response is expressed in terms of yield strength and Vicker’s hardness or hardness. These quantities are attributed to multiple contributions, such as grain boundary strengthening, order strengthening and mixtures of fcc and bcc regions and additional phases (viz. Laves) and precipitates. The initial yield stress computed by models must only be compared to measured hardness values with additional knowledge of the work hardening rates [71]. Thus, smart experiments must be designed to control other variables for an accurate account of the respective strengthening mechanisms, although is not very clear how to control the other contributions. The required breakthroughs include the development of accurate models for choosing optimal composition, high throughput synthesis and processing methods for HEAs and efficient experimental screening tools for structure-sensitive properties. Miracle et al. [92] elucidated a strategy to rapidly evaluate structural materials, especially HEAs, using high-throughput experiments. The authors noted that the order in which high throughput evaluation is conducted matters a great deal. For instance, stage 0 includes phase diagrams of candidate alloys obtained by CALPHAD to identify alloys with a good potential as structural materials. Stage 1 includes high-throughput experiments to evaluate properties that depend primarily on composition and that are insensitive to microstructures. Such properties are elastic modulus, density, thermal conductivity, coefficient of thermal expansion and environmental resistance. This stage allows the screening of compositions to give material libraries with compositions of interest. Stage 2 includes high throughput experiments to evaluate properties that depend strongly on microstructure. These properties include tensile strength, tensile ductility, fracture toughness and creep and fatigue resistance. However, rapid experiments to evaluate these properties are not adequately developed. This stage uses the material libraries of fixed composition (developed from previous stage) and controlled microstructure gradients. The currently developed equilibrium phase diagram via CALPHAD enables the calculation of the type of phases present and the transformation temperature; however, it does not establish the essential microstructural features such as phase size, shape, and distribution. These features, as noted by Miracle et al. [92], depend on nucleation mechanisms, diffusion rates, interfacial energies and elastic properties of the phases. Alas, the authors further noted that accurate high throughput models that can predict these microstructural features are generally not yet available. Complete fundamental understanding of underlying structural features must be achieved to engineer processing conditions that will maximize their effects. Ma [93] elucidated that there is a tendency towards local chemical orders and lattice-strain-induced modulation in HEAs, because multiple elements interact in a complex way to lower enthalpy. Therefore, such fluctuations in local chemical and structural disorder need statistical predictions for their spatial distribution because they influence dislocation energetics and configurations. A solution-strengthening model purely based on lattice friction hardening is not valid for most HEAs [65]. Several HEAs with a composition of interest must then be assessed at all possible conditions to provide reliable experiment data. In addition, their mass production and processing must be economical for industries. George et al. [31] noted that the development of HEAs into the market do not rely only on the price of alloying elements, but also on the feasibility of scrap recovery and recycling. Thus, all these considerations will facilitate transition from commercially used superalloys to theoretically understood HEAs for application purposes. However, the field is still in its incipient stage and more experimental investigation guided by simple yet detailed and reliable theoretical models are essential.

## 6. Conclusions

The computational and theoretical approaches unquestionably represent an efficient route in the design of HESAs, considering the tremendous composition domain possible in this alloy system. Of the HESAs studied, none can be shown unequivocally to be superior to Ni-based superalloys in high temperature mechanical properties. This may be attributed to a lack of full exhaustion of HESAs’ possible composition and microstructural features at several length scales, in part caused by the partial understanding of fundamental mechanistic behaviors in HESAs. It is necessary that characterization and simulation techniques be advanced to efficiently probe fundamental material properties such as solute-dislocation energies, accurate solute misfit volumes at composition of interest and dislocation core structure, to enable robust models specifically of solute solution strengthening. Of all strengthening mechanisms, solute solution strengthening is regarded as the most potent mechanism in HEAs, owing to the complexity introduced by multiple solutes, at their multifarious, with almost equal concentration. The development of HESAs since their birth have been dominated by experimental evaluations, motivated to mimic superalloys FCC + L12 interphase structures, and thus take advantage of high temperature strength induced by precipitation hardening and in addition, utilise HEAs’ assumed intrinsic core effects, i.e., high configurational entropy, lattice distortion, sluggish diffusion and cocktail effect. However, continuing in that direction is time consuming, let alone wasteful in terms of resources. The reliable computational methods to screen composition of interest, followed by rapid high-throughput alloy evaluations, will reduce time and usage of resources for HEAs/HESAs research. Powder metallurgy appears to be an efficient route for the rapid fabrication of selected HEAs that shows superior combination of properties, owing to its versatility, short time processing and ease of microstructural control. Nonetheless, currently reliable CALPHAD techniques cannot uncover metastable, microstructural constituents and transient phases in HEAs, and hence no processing parameters can be completely optimised because there is no phase equilibrium to refer to. The critical aspect of solute solution strengthening in HEAs is the prevalent local fluctuations in chemical composition, which might affect the spatial configuration of crystallographic defects. If such a local ordering of elements on the structure of alloys can be predicted with their distribution, it might be possible to trigger simultaneous and diverse strengthening that is impossible in dilute alloys. The clear measures of local chemical order propensity in HESAs, dislocation core structures and solute-dislocation energy in detail, will enable the property-oriented design of HESAs.

## Figures and Tables

**Table 2:** Equations modelling precipitation hardening of shearing mechanism [63].

Particle-Matrix Coherency (Δσ_cs_)	Modulus Mismatch (Δσ_ms_)	Atomic Ordering (Δσ_os_)
M·αεG·ε32rf0.5Gb12	M·0.005ΔG322fG12rb3m2−1	$M\cdot 0.81\frac{\gamma_{\mathrm{APB}}}{2b}{\left(\frac{3\pi f}{8}\right)}^{\frac{1}{2}}$

**Table 3:** Shows the processing route, phases and mechanical properties of HESAs extracted from the studies. BC-Bridgemann casting, AC-As-cast, CR-Cold-rolled, HIP-Hot-Isostatic pressing, SX-Single crystal, MA- and SPS-Mechanical alloying and spark plasma sintering, T-Tension, C-Compression, έ-strain rate, T (°C) temperature, σ_y_-Yield strength, UTS-Ultimate tensile strength, ε_f_-elongation to fracture, Hv-Hardness in Vickers, Ref.-references.

Alloy	Processing	Phases	ε˙(s−1)	Test	T (°C)	σ_y_ (MPa)	UTS (MPa)	εf (%)	Hv	Ref.
Al0.3CoCrFeNi	BC	SX [001]	4 × 10 − 4	T	23	185	399	~80	-	[30]
	AC	FCC + L12	4 × 10 − 4		23	224 ± 51	434 ± 94	48 ± 10	-	
Al0.3CrCuFeNi2	AC	FCC + L12 + Cu-rich phase	-	-	-	-	-	-	171 ± 05	[4]
	550 °C/150 h		-	-	-	-	-	-	294 ± 09	
	700 °C/50 h		-	-	-	-	-	-	273 ± 11	
Al0.3CoCrFeNi	AC	FCC + L12	-	-	-	-	-	-	152 ± 11	[4]
	550 °C/150 h	FCC + B2	-	-	-	-	-	-	216 ± 07	
	700 °C/50 h		-	-	-	-	-	-	259 ± 02	
Al0.5CoCrCu0.5FeNi2	CR 43%, 1100 °C/24	FCC + L12	1 × 10 − 3	T	23	360 ± 100	639 ± 5	3.4 ± 0.4	-	[30]
Al0.5CoCrCu0.5FeNi2	AC	FCC + L12	3.3 × 10 − 3	T	RT	357	459	9	275	[48]
	700 °C/5 h					365	365	0.1	-	
	1150 °C/5 h					215	489	39	-	
	AC				500	315 ± 12	334 ± 1.0	0.7 ± 0.3	-	
	700 °C/5 h					310 ± 2.0	310 ± 2.0	<0.02	-	
	1150 °C/5 h					215 ± 11	248 ± 10	6.0 ± 3.0	-	
Al0.5CoCrCuFeNi	1000 °C/6h, CR 84%	FCC + L12	1 × 10 − 3	T	23	1248	1344	7.6	-	[30]
Al0.2CrCoCu0.2FeNi2	700 °C/20 h	FCC + L12	1.7 × 10 − 3	T	RT	719	1048	30.4	-	[26]
	800 °C/1 h					460	732	31.7	-	
Al0.7Co1.7Cr0.5FeNi2.4Ti0.4	1220 °C/20 h–900 °C/5 h	FCC + L12	0.83 × 10 − 4	T	RT	786	568	12	-	[25]
					600	674	501	26	-	
					700	702	487	18	-	
					800	672	535	27	-	
					1000	148	–	92	-	
	1220 °C/20 h–900 °C/50 h	FCC + L12	0.83 × 10 − 4	T	RT	1039	596	20	-	
					600	809	509	27	-	
					700	624	486	11	-	
					800	687	581	9	-	
Al8Co17Cr14Cu8Fe17Ni34.8W0.1Mo0.1Ti1	AC	FCC + L12	-	-	-	-	-	-	225	[49]
	700 °C/24 h	FCC + L12	-	-	-	-	-	-	-	
(FeCoNiCr)94Ti2Al4	CR 70%, 650 °C/4 h	FCC + L12 + L21	1 × 10 − 3	T	RT	1005	1273	17		[8]
	CR 30%, 1000 °C/2h, 800 °C/18 h	FCC + L12 + L21		T	RT	645	1094	39		
Co1.5CrFeNi1.5Ti0.5	MA & SPS	FCC + η + L12	0.25 × 10 − 4	T	After SPS	1289 ± 6.5	1569 ± 11.5	6.15 ± 0.59	472 ± 27	[85]
					700 °C	1388 ± 36.8	1661 ± 13.5	4.35 ± 0.19	515 ± 19	
					900 °C	886 ± 3.5	1236 ± 0.5	9.73 ± 0.04	386 ± 13	
					1100 °C	1048	1467	14.43	384 ± 88	
Co1.5CrFeNi1.5Ti	AC	FCC + L12	-	-	-	-	-	-	654	[47]
Ni45(FeCoCr)40(AlTi)15	AC	FCC + L12	1 × 10 − 3	C	RT	1110	-	-	-	[32]
					750	855	1569	-	-	
					850	796	951	-	-	
					950	560	597	-	-	
Al0.2Co1.5CrFeNi1.5Ti0.3	1150 °C/3 h	FCC + L12	1 × 10 − 3	T	RT	540	917	50	-	[24]
	1150 °C/3 h + 800 °C/5 h					760	1160	40	-	
CoFeNiSi0.5	AC	FCC + Ni3Si	2 × 10 − 4	C	RT	476	2250 − 2500	40 − 50	287	[87]
CoFeNiSi0.75	AC	FCC + Ni3Si	2 × 10 − 4	C	RT	1301	2000	0 − 5	570	
Al0.3Cr0.5Mn0.6FeNi0.4	AC	BCC + B2 + Minor FCC	10 − 4	C	25	750	880	2.5	420 ± 10	[82]
					400	640	900	20	-	
					500	515	715	42	-	
					600	310	404	55	-	
Al40(CoCrCuFeMnNiTiV)60	AC	BCC + B2	1 × 10−4	C	RT	1461	1461	<1	-	[88]
AlMo0.5NbTa0.5TiZr	HIP 1400 °C/207 MPa/2 h, 1400 °C/24 h	BCC + B2	10 − 3	C	23	2000	880	-	-	[89]
					800	1597	900	-	-	
					1000	745	715	-	-	
					1200	255	405	-	-	
NbTiVZr	AC	BCC + B2	2 × 10 − 4	C	RT	1105	-	>50	335	[90]
TiZrNbVMo1.3.	AC	BCC + B2	2 × 10 − 4	C	RT	1496	-	30	-	[91]
TiZrNbVMo1.5	AC	BCC + B2	2 × 10 − 4		RT	1603	-	20	-	
TiZrNbVMo1.7	AC	BCC + B2	2 × 10 − 4		RT	1645	-	15	-	
TiZrNbVMo2.0	AC	BCC + B2	2 × 10 − 4		RT	1765	-	12	-	

## Data Availability

The data presented in this study are available in [30,74,90,94].

## Abbreviations

Abbreviation	Definition
HEAs	High entropy alloys
HESAs	High entropy superalloys
FCC	Face-centered cubic
BCC	Body-centered cubic
HCP	Hexagonal close-packed
VEC	Valence electron concentration
CALPHAD	Calculations of phase diagram
TWIP	Twinning-induced plasticity
XRD	X-ray diffraction
EDX	Energy dispersive X-ray
EBSD	Electron backscatter diffraction
SAED	Selected area electron diffraction
TEM	Transmission electron microscope
SEM	Scanning electron microscope
HAADF	High-angle annular dark field
APT	Atom probe tomography
3d-TM	3d-transition-metals
L12	Ordered FCC structure
A1	Disordered FCC structure
A2	Disordered BCC structure
B2	Ordered BCC structure
CCA	Complex concentrated alloys
MA	Mechanical alloying
SPS	Spark plasma sintering

## References

[1] Yeh J.-W., Chen S.-K., Lin S.-J., Gan J.-Y., Chin T.-S., Shun T.-T., Tsau C.-H., Chang S.-Y. (2004). Nanostructured High-Entropy Alloys with Multiple Principal Elements: Novel Alloy Design Concepts and Outcomes. Adv. Eng. Mater..

[2] Cantor B., Chang I., Knight P., Vincent A. (2004). Microstructural development in equiatomic multicomponent alloys. Mater. Sci. Eng. A.

[3] Cheng H., Wang H.Y., Xie Y.C., Tang Q.H., Dai P.Q. (2017). Controllable fabrication of a carbide-containing FeCoCrNiMn high-entropy alloy: Microstructure and mechanical properties. Mater. Sci. Technol..

[4] Gwalani B., Soni V., Choudhuri D., Lee M., Hwang J., Nam S., Ryu H.J., Hong S.H., Banerjee R. (2016). Stability of ordered L12 and B2 precipitates in face centered cubic based high entropy alloys—Al0.3CoFeCrNi and Al0.3CuFeCrNi2. Scr. Mater..

[5] Slone C., George E., Mills M. (2019). Elevated temperature microstructure evolution of a medium-entropy CrCoNi superalloy containing Al,Ti. J. Alloys Compd..

[6] Stepanov N., Shaysultanov D., Tikhonovsky M., Zherebtsov S. (2018). Structure and high temperature mechanical properties of novel non-equiatomic Fe-(Co, Mn)-Cr-Ni-Al-(Ti) high entropy alloys. Intermetallics.

[7] Sathiyamoorthi P., Basu J., Kashyap S., Pradeep K., Kottada R.S. (2017). Thermal stability and grain boundary strengthening in ultrafine-grained CoCrFeNi high entropy alloy composite. Mater. Des..

[8] He J., Wang H., Wu Y., Liu X., Mao H., Nieh T., Lu Z. (2016). Precipitation behavior and its effects on tensile properties of FeCoNiCr high-entropy alloys. Intermetallics.

[9] Praveen S., Anupam A., Tilak R., Kottada R.S. (2018). Phase evolution and thermal stability of AlCoCrFe high entropy alloy with carbon as unsolicited addition from milling media. Mater. Chem. Phys..

[10] Shivam V., Basu J., Pandey V.K., Shadangi Y., Mukhopadhyay N. (2018). Alloying behaviour, thermal stability and phase evolution in quinary AlCoCrFeNi high entropy alloy. Adv. Powder Technol..

[11] Yeh A.C., Tsao T.K., Chang Y.J., Chang K.C., Yeh J.W., Chiou M.S., Jian S.R., Kuo C.M., Wang W.R., Murakami H. (2015). Developing New Type of High Temperature Alloys–High Entropy Superalloys. Int. J. Metall. Mater. Eng..

[12] Keil T., Bruder E., Durst K. (2019). Exploring the compositional parameter space of high-entropy alloys using a diffusion couple approach. Mater. Des..

[13] Wang P., Cai H., Zhou S., Xu L. (2017). Processing, microstructure and properties of Ni1.5CoCuFeCr0.5−xVx high entropy alloys with carbon introduced from process control agent. J. Alloys Compd..

[14] Kong T., Kang B., Ryu H.J., Hong S.H. (2020). Microstructures and enhanced mechanical properties of an oxide dispersion-strengthened Ni-rich high entropy superalloy fabricated by a powder metallurgical process. J. Alloys Compd..

[15] Fang S., Chen W., Fu Z. (2014). Microstructure and mechanical properties of twinned Al0.5CrFeNiCo0.3C0.2 high entropy alloy processed by mechanical alloying and spark plasma sintering. Mater. Des..

[16] Yim D., Sathiyamoorthi P., Hong S.-J., Kim H.S. (2018). Fabrication and mechanical properties of TiC reinforced CoCrFeMnNi high-entropy alloy composite by water atomization and spark plasma sintering. J. Alloys Compd..

[17] Masemola K., Popoola P., Malatji N. (2020). The effect of annealing temperature on the microstructure, mechanical and electrochemical properties of arc-melted AlCrFeMnNi equi-atomic High entropy alloy. J. Mater. Res. Technol..

[18] Munitz A., Kaufman M., Nahmany M., Derimow N., Abbaschian R. (2018). Microstructure and mechanical properties of heat treated Al1.25CoCrCuFeNi high entropy alloys. Mater. Sci. Eng. A.

[19] Senkov O.N., Isheim D., Seidman D.N., Pilchak A.L. (2016). Development of a Refractory High Entropy Superalloy. Entropy.

[20] Detrois M., Jablonski P.D., Antonov S., Li S., Ren Y., Tin S., Hawk J.A. (2019). Design and thermomechanical properties of a γʹ precipitate-strengthened Ni-based superalloy with high entropy γ matrix. J. Alloys Compd..

[21] Waseem O.A., Ryu H.J. (2017). Powder Metallurgy Processing of a WxTaTiVCr High-Entropy Alloy and Its Derivative Alloys for Fusion Material Applications. Sci. Rep..

[22] Dong Y., Yao Z., Huang X., Du F., Li C., Chen A., Wu F., Cheng Y., Zhang Z. (2020). Microstructure and mechanical properties of AlCoxCrFeNi3-x eutectic high-entropy-alloy system. J. Alloys Compd..

[23] Tsao T.-K., Yeh A.-C., Kuo C.-M., Murakami H. (2016). On The Superior High Temperature Hardness of Precipitation Strengthened High Entropy Ni-Based Alloys. Adv. Eng. Mater..

[24] Ming K., Bi X., Wang J. (2018). Realizing strength-ductility combination of coarse-grained Al0.2Co1.5CrFeNi1.5Ti0.3 alloy via nano-sized, coherent precipitates. Int. J. Plast..

[25] Daoud H.M., Manzoni A., Wanderka N., Glatzel U. (2015). High-Temperature Tensile Strength of Al10Co25Cr8Fe15Ni36Ti6 Compositionally Complex Alloy (High-Entropy Alloy). JOM.

[26] Wang Z., Zhou W., Fu L., Wang J., Luo R., Han X., Chen B., Wang X. (2017). Effect of coherent L12 nanoprecipitates on the tensile behavior of a fcc-based high-entropy alloy. Mater. Sci. Eng. A.

[27] Zheng F., Zhang G., Chen X., Yang X., Yang Z., Li Y., Li J. (2020). A new strategy of tailoring strength and ductility of CoCrFeNi based high-entropy alloy. Mater. Sci. Eng. A.

[28] Niu S., Kou H., Guo T., Zhang Y., Wang J., Li J. (2016). Strengthening of nanoprecipitations in an annealed Al0.5CoCrFeNi high entropy alloy. Mater. Sci. Eng. A.

[29] Praveen S., Kim H.S. (2017). High-Entropy Alloys: Potential Candidates for High-Temperature Applications—An Overview. Adv. Eng. Mater..

[30] Miracle D., Senkov O. (2017). A critical review of high entropy alloys and related concepts. Acta Mater..

[31] George E.P., Raabe D., Ritchie R.O. (2019). High-entropy alloys. Nat. Rev. Mater..

[32] Zhang W., Liaw P.K., Zhang Y. (2018). Science and technology in high-entropy alloys. Sci. China Mater..

[33] Chen H.-L., Mao H., Chen Q. (2017). Database development and Calphad calculations for high entropy alloys: Challenges, strategies, and tips. Mater. Chem. Phys..

[34] Wu M., Wang S., Huang H., Shu D., Sun B. (2019). CALPHAD aided eutectic high-entropy alloy design. Mater. Lett..

[35] Wang W., Chen H.-L., Larsson H., Mao H. (2019). Thermodynamic constitution of the Al–Cu–Ni system modeled by CALPHAD and ab initio methodology for designing high entropy alloys. Calphad.

[36] Manzoni A.M., Glatzel U. (2018). New multiphase compositionally complex alloys driven by the high entropy alloy approach. Mater. Charact..

[37] Mishra R.S., Haridas R.S., Agrawal P. (2021). High entropy alloys—Tunability of deformation mechanisms through integration of compositional and microstructural domains. Mater. Sci. Eng. A.

[38] Gao M.C., Yeh J.W., Liaw P.K., Zhang Y. (2016). High-Entropy Alloys.

[39] Miracle D.B., Tsai M.-H., Senkov O.N., Soni V., Banerjee R. (2020). Refractory high entropy superalloys (RSAs). Scr. Mater..

[40] Senkov O.N., Woodward C., Miracle D.B. (2014). Microstructure and Properties of Aluminum-Containing Refractory High-Entropy Alloys. JOM.

[41] Senkov O., Senkova S., Woodward C. (2014). Effect of aluminum on the microstructure and properties of two refractory high-entropy alloys. Acta Mater..

[42] Whitfield T.E., Pickering E.J., Owen L.R., Senkov O.N., Miracle D.B., Stone H.J., Jones N.G. (2020). An assessment of the thermal stability of refractory high entropy superalloys. J. Alloys Compd..

[43] Soni V., Senkov O.N., Gwalani B., Miracle D.B., Banerjee R. (2018). Microstructural Design for Improving Ductility of An Initially Brittle Refractory High Entropy Alloy. Sci. Rep..

[44] Senkov O.N., Miracle D.B., Chaput K.J., Couzinie J.-P. (2018). Development and exploration of refractory high entropy alloys—A review. J. Mater. Res..

[45] Soni V., Senkov O., Couzinie J.-P., Zheng Y., Gwalani B., Banerjee R. (2019). Phase stability and microstructure evolution in a ductile refractory high entropy alloy Al10Nb15Ta5Ti30Zr40. Materialia.

[46] Yeh A.-C., Chang Y.-J., Tsai C.-W., Wang Y.-C., Yeh J.-W., Kuo C.-M. (2013). On the Solidification and Phase Stability of a Co-Cr-Fe-Ni-Ti High-Entropy Alloy. Met. Mater. Trans. A.

[47] Chuang M., Tsai M., Wang W., Lin S., Yeh J. (2011). Microstructure and wear behavior of AlxCo1. 5CrFeNi1. 5Tiy high-entropy alloys. Acta Mater..

[48] Daoud H.M., Manzoni A., Volkl R., Wanderka N., Glatzel U. (2013). Microstructure and Tensile Behavior of Al8Co17Cr17Cu8Fe17Ni33 (at.%) High-Entropy Alloy. JOM.

[49] Manzoni A.M., Daoud H.M., Voelkl R., Glatzel U., Wanderka N. (2015). Influence of W, Mo and Ti trace elements on the phase separation in Al8Co17Cr17Cu8Fe17Ni33 based high entropy alloy. Ultramicroscopy.

[50] Pickering E., Stone H., Jones N. (2015). Fine-scale precipitation in the high-entropy alloy Al0.5CrFeCoNiCu. Mater. Sci. Eng. A.

[51] Tsao T.-K., Yeh A.-C., Murakami H. (2017). The Microstructure Stability of Precipitation Strengthened Medium to High Entropy Superalloys. Met. Mater. Trans. A.

[52] Zhao Y., Chen H., Lu Z., Nieh T. (2018). Thermal stability and coarsening of coherent particles in a precipitation-hardened (NiCoFeCr)94Ti2Al4 high-entropy alloy. Acta Mater..

[53] Kang B., Kong T., Ryu H.J., Hong S.H. (2019). The outstanding tensile strength of Ni-rich high entropy superalloy fabricated by powder metallurgical process. Mater. Chem. Phys..

[54] Shafiee A., Moon J., Kim H.S., Jahazi M., Nili-Ahmadabadi M. (2019). Precipitation behaviour and mechanical properties of a new wrought high entropy superalloy. Mater. Sci. Eng. A.

[55] Dieter G.E. (1988). Mechanical Metallurgy, SI Metric.

[56] Varvenne C., Leyson G., Ghazisaeidi M., Curtin W. (2017). Solute strengthening in random alloys. Acta Mater..

[57] Varvenne C., Luque A., Curtin W.A. (2016). Theory of strengthening in fcc high entropy alloys. Acta Mater..

[58] Toda-Caraballo I., Rivera-Díaz-Del-Castillo P.E. (2015). Modelling solid solution hardening in high entropy alloys. Acta Mater..

[59] Gil Coury F., Wilson P., Clarke K., Kaufman M.J., Clarke A.J. (2019). High-throughput solid solution strengthening characterization in high entropy alloys. Acta Mater..

[60] LaRosa C.R., Shih M., Varvenne C., Ghazisaeidi M. (2019). Solid solution strengthening theories of high-entropy alloys. Mater. Charact..

[61] Wu Z., Gao Y., Bei H. (2016). Thermal activation mechanisms and Labusch-type strengthening analysis for a family of high-entropy and equiatomic solid-solution alloys. Acta Mater..

[62] Leyson G., Curtin W. (2013). Friedel vs. Labusch: The strong/weak pinning transition in solute strengthened metals. Philos. Mag..

[63] He J., Wang H., Huang H., Xu X., Chen M., Wu Y., Liu X., Nieh T., An K., Lu Z. (2016). A precipitation-hardened high-entropy alloy with outstanding tensile properties. Acta Mater..

[64] Li J., Gao B., Wang Y., Chen X., Xin Y., Tang S., Liu B., Liu Y., Song M. (2019). Microstructures and mechanical properties of nano carbides reinforced CoCrFeMnNi high entropy alloys. J. Alloys Compd..

[65] Basu I., De Hosson J.T. (2020). Strengthening mechanisms in high entropy alloys: Fundamental issues. Scr. Mater..

[66] Bracq G., Laurent-Brocq M., Varvenne C., Perrière L., Curtin W., Joubert J.-M., Guillot I. (2019). Combining experiments and modeling to explore the solid solution strengthening of high and medium entropy alloys. Acta Mater..

[67] Leyson G.P.M., Curtin W.A. (2016). Solute strengthening at high temperatures. Model. Simul. Mater. Sci. Eng..

[68] Toda-Caraballo I. (2017). A general formulation for solid solution hardening effect in multicomponent alloys. Scr. Mater..

[69] Yoshida S., Ikeuchi T., Bhattacharjee T., Bai Y., Shibata A., Tsuji N. (2019). Effect of elemental combination on friction stress and Hall-Petch relationship in face-centered cubic high/medium entropy alloys. Acta Mater..

[70] Okamoto N., Yuge K., Tanaka K., Inui H., George E. (2016). Atomic displacement in the CrMnFeCoNi high-entropy alloy—A scaling factor to predict solid solution strengthening. AIP Adv..

[71] George E., Curtin W., Tasan C. (2019). High entropy alloys: A focused review of mechanical properties and deformation mechanisms. Acta Mater..

[72] Walbrühl M., Linder D., Ågren J., Borgenstam A. (2017). Modelling of solid solution strengthening in multicomponent alloys. Mater. Sci. Eng. A.

[73] Varvenne C., Curtin W.A. (2017). Strengthening of high entropy alloys by dilute solute additions: CoCrFeNiAl x and CoCrFeNiMnAl x alloys. Scr. Mater..

[74] Yin B., Maresca F., Curtin W. (2020). Vanadium is an optimal element for strengthening in both fcc and bcc high-entropy alloys. Acta Mater..

[75] Gladman T. (1999). Precipitation hardening in metals. Mater. Sci. Technol..

[76] Abd El-Aty A., Xu Y., Guo X., Zhang S.H., Ma Y., Chen D. (2018). Strengthening mechanisms, deformation behavior, and anisotropic mechanical properties of Al-Li alloys: A review. J. Adv. Res..

[77] Tsao T.-K., Yeh A.-C., Kuo C.-M., Kakehi K., Murakami H., Yeh J.-W., Jian S.-R. (2017). The High Temperature Tensile and Creep Behaviors of High Entropy Superalloy. Sci. Rep..

[78] Tsao T.K., Yeh A.C., Kuo C.M., Murakami H. (2016). High temperature oxidation and corrosion properties of high entropy superalloys. Entropy..

[79] Microstructures and Properties of High Entropy Alloys, Scientific Figure on Researchgate. https://www.researchgate.net/figure/A-schematic-diagram-of-the-arc-melting-method-99_fig17_259887707.

[80] Senkov O., Couzinie J.-P., Rao S., Soni V., Banerjee R. (2020). Temperature dependent deformation behavior and strengthening mechanisms in a low density refractory high entropy alloy Al10Nb15Ta5Ti30Zr40. Materialia.

[81] Journal of Material Science. Adopted from: Flexible Scanner-Based Laser Surface Treatment,” Scientific Figure on ResearchGate. https://www.researchgate.net/figure/Principle-of-the-laser-cladding-process_fig1_239028642.

[82] Shaysultanov D., Salishchev G., Ivanisenko Y., Zherebtsov S., Tikhonovsky M., Stepanov N. (2017). Novel Fe36Mn21Cr18Ni15Al10 high entropy alloy with bcc/B2 dual-phase structure. J. Alloys Compd..

[83] Moravcikova-Gouvea L., Moravcik I., Omasta M., Veselý J., Cizek J., Minárik P., Cupera J., Záděra A., Jan V., Dlouhy I. (2019). High-strength Al0.2Co1.5CrFeNi1.5Ti high-entropy alloy produced by powder metallurgy and casting: A comparison of microstructures, mechanical and tribological properties. Mater. Charact..

[84] Fu Z., Chen W., Wen H., Zhang D.Z., Chen Z., Zheng B., Zhou Y., Lavernia E.J. (2016). Microstructure and strengthening mechanisms in an FCC structured single-phase nanocrystalline Co25Ni25Fe25Al7.5Cu17.5 high-entropy alloy. Acta Mater..

[85] Moravcik I., Cizek J., Zapletal J., Kovacova Z., Vesely J., Minarik P., Kitzmantel M., Neubauer E., Dlouhy I. (2017). Microstructure and mechanical properties of Ni1,5Co1,5CrFeTi0,5 high entropy alloy fabricated by mechanical alloying and spark plasma sintering. Mater. Des..

[86] Ma S., Zhang S., Qiao J., Wang Z., Gao M., Jiao Z., Yang H., Zhang Y. (2014). Superior high tensile elongation of a single-crystal CoCrFeNiAl0.3 high-entropy alloy by Bridgman solidification. Intermetallics.

[87] Zuo T., Li R., Ren J., Zhang A. (2014). Effects of Al and Si addition on the structure and properties of CoFeNi equal atomic ratio alloy. J. Magn. Magn. Mater..

[88] Zhou Y., Zhang Y., Wang Y., Chen G. (2007). Microstructure and compressive properties of multicomponent Alx(TiVCrMnFeCoNiCu)100−x high-entropy alloys. Mater. Sci. Eng. A.

[89] Senkov O., Senkova S., Woodward C., Miracle D. (2013). Low-density, refractory multi-principal element alloys of the Cr–Nb–Ti–V–Zr system: Microstructure and phase analysis. Acta Mater..

[90] Couzinié J.-P., Senkov O., Miracle D., Dirras G. (2018). Comprehensive data compilation on the mechanical properties of refractory high-entropy alloys. Data Brief.

[91] Wu Y., Cai Y., Chen X., Wang T., Si J., Wang L., Wang Y., Hui X. (2015). Phase composition and solid solution strengthening effect in TiZrNbMoV high-entropy alloys. Mater. Des..

[92] Miracle D., Majumdar B., Wertz K., Gorsse S. (2017). New strategies and tests to accelerate discovery and development of multi-principal element structural alloys. Scr. Mater..

[93] Ma E. (2020). Unusual dislocation behavior in high-entropy alloys. Scr. Mater..

[94] Chen J., Zhou X., Wang W., Liu B., Liu B., Yang W., Xu D., Liu Y. (2018). A review on fundamental of high entropy alloys with promising high–temperature properties. J. Alloys Compd..

